# Synthesis and Thrombin, Factor Xa and U46619 Inhibitory Effects of Non-Amidino and Amidino *N*^2^-Thiophenecarbonyl- and *N*^2^-Tosylanthranilamides

**DOI:** 10.3390/ijms18061144

**Published:** 2017-05-31

**Authors:** Soo Hyun Lee, Wonhwa Lee, ThiHa Nguyen, Il Soo Um, Jong-Sup Bae, Eunsook Ma

**Affiliations:** 1 College of Pharmacy, Catholic University of Daegu, Hayang-ro 13-13, Hayang-eup, Gyeongsan-si, Gyeongbuk 712-702, Korea; Soohyunlee@cu.ac.kr (S.H.L.); Nguyenthiha@gmail.com (T.N.); isu404777@cu.ac.kr (I.S.U.); 2 College of Pharmacy, CMRI, Research Institute of Pharmaceutical Sciences, BK21 Plus KNU Multi-Omics Based Creative Drug Research Team, Kyungpook National University, 80 Daehak-ro, Buk-gu, Daegu 41566, Korea; ByWonhwalee@gmail.com

**Keywords:** *N*^2^-Arylcarbonyl/sulfonylanthranilamides, prothrombin time, activated partial thromboplastin time, Thrombin, Factor Xa, U46619

## Abstract

Thrombin (factor IIa) and factor Xa (FXa) are key enzymes at the junction of the intrinsic and extrinsic coagulation pathways and are the most attractive pharmacological targets for the development of novel anticoagulants. Twenty non-amidino *N*^2^-thiophencarbonyl- and *N*^2^-tosyl anthranilamides **1**–**20** and six amidino *N*^2^-thiophencarbonyl- and *N*^2^-tosylanthranilamides **21**–**26** were synthesized to evaluate their activated partial thromboplastin time (aPTT) and prothrombin time (PT) using human plasma at a concentration of 30 µg/mL in vitro. As a result, compounds **5**, **9**, and **21**–**23** were selected to study the further antithrombotic activity. The anticoagulant properties of **5**, **9**, and **21**–**23** significantly exhibited a concentration-dependent prolongation of in vitro PT and aPTT, in vivo bleeding time, and ex vivo clotting time. These compounds concentration-dependently inhibited the activities of thrombin and FXa and inhibited the generation of thrombin and FXa in human endothelial cells. In addition, data showed that **5**, **9**, and **21**–**23** significantly inhibited thrombin catalyzed fibrin polymerization and mouse platelet aggregation and inhibited platelet aggregation induced by U46619 in vitro and ex vivo. Among the derivatives evaluated, *N*-(3′-amidinophenyl)-2-((thiophen-2′′-yl)carbonylamino)benzamide (**21**) was the most active compound.

## 1. Introduction

Thromboembolic disorders, such as cerebral thrombosis, deep vein thrombosis, unstable angina, myocardial infarction, and thromboembolic stroke; are one of the major disorders that cause morbidities and mortalities worldwise [1]. Several anticoagulants, such as heparins and vitamin K antagonists, have known to be effective in the treatment and prevention of venous thromboembolic diseases [2]. However, there are significant shortcomings, such as monitoring, inconvenience of drug administration, bleeding for heparins, and such as considerable drug and food interactions for vitamin K antagonists, therefore their clinical application was restricted [3].

Thrombin (factor IIa, FIIa) and factor Xa (FXa) occupy central positions in the blood coagulation cascade and play prominent role in various thromboembolic complications. Thrombin promotes blood clot formation by the conversion of fibrinogen to insoluble fibrin and activating platelets [4]. Inhibition of thrombin attenuates formation of fibrin, reduces thrombin generation, and may limit platelet aggregation. FXa is a trypsin like serine protease which plays a pivotal role in the sequence of blood coagulation events. By directly binding to FXa active site, FXa inhibitors lead to interruption of the intrinsic and extrinsic coagulation cascade pathways and thus to inhibition of thrombin formation and thrombus development.

Heparin inhibits both thrombin and factor Xa indirectly via complex formation with modulation of the activity of the serine protease inhibitor antithrombin III [5]. Fondaparinux sodium (Arixtra^®^), synthetic heparin pentasaccharide, acts as antithrombin III (AT-III) mediated selective inhibition of FXa, distinct from heparin. Its pharmacokinetic properties is nearly complete bioavailability subcutaneously to allow for a simple, fixed-dose, a prolonged half-life, without the need for monitoring [6,7]. Unexpectedly, fondaparinux sodium is inconvenient for long-term use and requires dose adjustment. If the therapeutic range and coagulation monitoring are necessary, it can be monitored with FXa levels by a chromogenic assay, particularly in hemorrhage and renal insufficiency, and it may also require the control of platelet contents [8].

The first synthetic direct thrombin inhibitor was ximelagatran, but it was not approved for medicinal use because of its hepatotoxicity. The direct thrombin inhibitor, dabigatran etexilate mesylate (Pradaxa^®^), argatroban, and synthetic 20-amino acid peptide, bivalirudin are approved for use on the market [9]. Apixaban (Eliquis^®^) and rivaroxaban (Xarelto^®^) are oral direct FXa inhibitors. But, while these anticoagulants carry similar side effects to warfarin, such as risk for gastrointestinal bleeding and intracranial hemorrhage, international normalized ratio (INR) and PT monitoring are not required.

The structures of thrombin and FXa show similarities in their active sites and for this reason, attempts have been made to develop novel synthetic compounds that exert a dual inhibition of the two main enzymes of the coagulation cascade. A combination of thrombin and FXa inhibitory activity in a single, synthetic, orally bioavailable, small molecular weight compound as a novel approach to antithrombotic therapy should therefore result in potent anticoagulant with potentially superior features over currently available therapies [10].

The first compounds to demonstrate dual inhibitor properties were BIBM 1015 and tanogitran (BIBT986) which belong to the methylbenzimidazole series [5]. Hexadecasaccharide (e.g., SR123781) [11,12] is a synthetic heparin mimetic that inhibits both FXa and thrombin via antithrombin III that was advanced to phase I clinical trials. This dual inhibitor demonstrated superior antithrombotic properties in three rat models of thrombosis following intravenous administration compared to selective factor Xa inhibitor, synthetic pentasaccharide fondaparinux (Arixtra^®^) [13]. In addition, novel anti-FXa pentasaccharides coupled to active site thrombin inhibitors to obtain dual inhibitory effects have also been reported [14,15]. However, problems with thrombin-rebound remain with these mimetics, which appear to result from the inability to inhibit clot-bound thrombin.

EP 217609 is a synthetic parenteral anticoagulant with a dual-action that combines a α-NAPAP analog (direct thrombin inhibitor) and a fondaparinux analog (indirect factor Xa inhibitor) in a single molecule without the complications of thrombin rebound [16]. In recent years several groups [17,18,19,20] have reported the development of dual FXa and thrombin inhibitors and proposed a stronger anticoagulant activity for such compounds compared to monoselective inhibitors.

Furthermore, some patients with cardiovascular disease have indications for anticoagulant and dual antiplatelet therapy. It is estimated that between 5 and 10 percent of patients scheduled for coronary artery stenting, and who thus require dual antiplatelet therapy, are receiving oral anticoagulant, most often for atrial fibrillation [21]. The concomitant use of dual antiplatelet therapy and oral anticoagulant is referred to as triple oral antithrombotic therapy (triple therapy).

We previously reported the synthesis and anticoagulant and antiplatelet activities of *N*^3^-aroylbenzamide derivatives. Two amidine compounds, *N*-(3′-amidino-, and *N*-(4′-amidinophenyl)-3-(thiophen-2′′-ylcarbonylamino)benzamide exhibited weak thrombin, FXa, and U46619 inhibitory activities [22]. Anthranilamide derivatives have been studied as FXa inhibitors [23,24,25,26], therefore anthranilic acid was considered as central ring of diamide to improve the activity. We now report the synthesis and evaluation of the thrombin, FXa, and U46619 inhibitory activities of non-amidino *N*^2^-thiophenecarbonyl- and *N*^2^-tosylanthranilamides and amidino *N*^2^-thiophenecarbonyl- and *N*^2^-tosylanthranilamides.

## 2. Results and Discussion

### 2.1. Chemistry

**Scheme 1:**
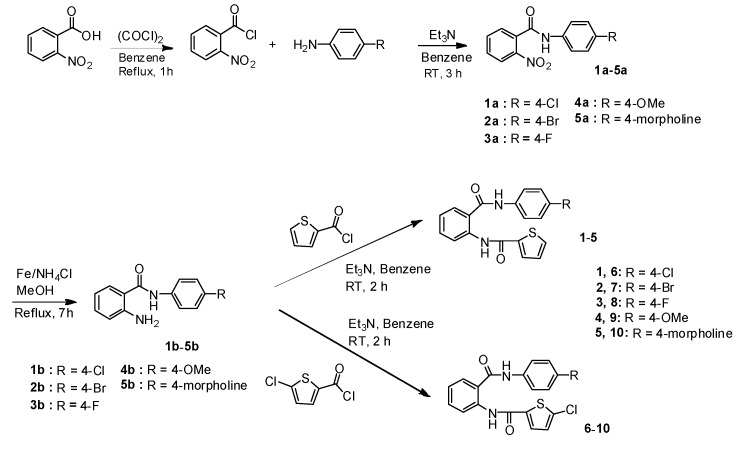
Synthesis of *N*^2^-(thiophenecarbonyl)anthranilamides derivatives **1**–**10**.

**Scheme 2:**
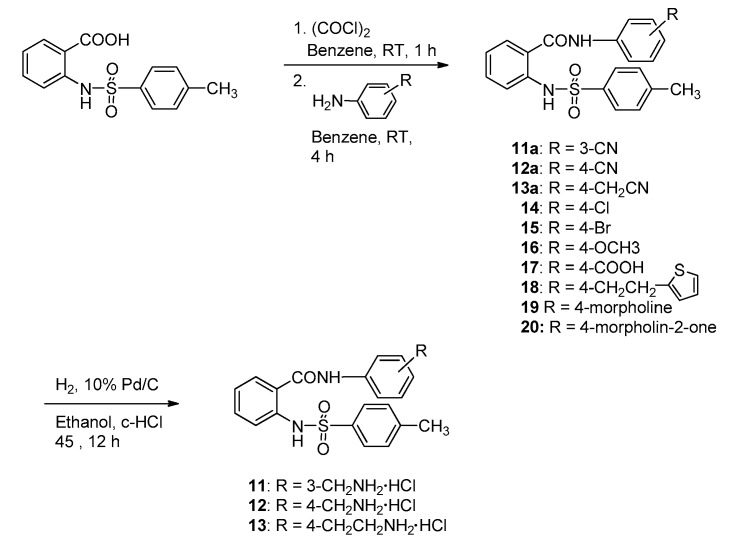
Synthetic procedures of *N*^2^-tosylanthranilamide derivatives **11**–**20**.

**Scheme 3:**
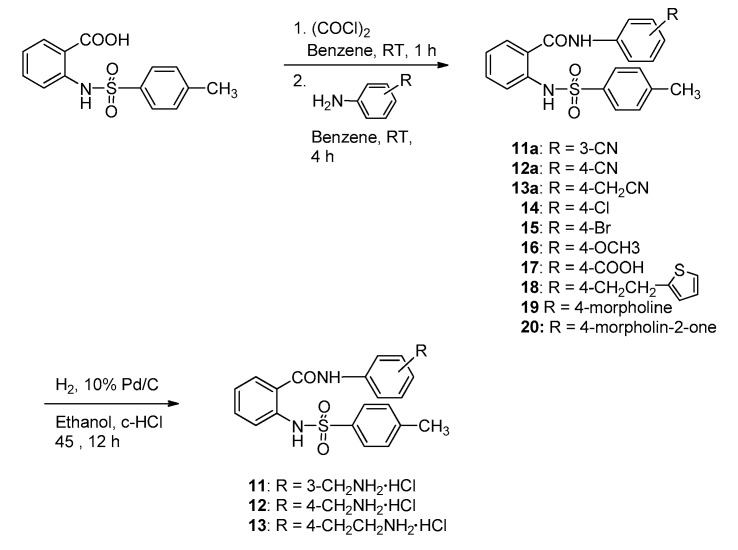
Synthetic procedures of amidino *N*^2^-(thiophenecarbonyl)anthranilamide derivatives (**21**–**23**).

**Scheme 4:**
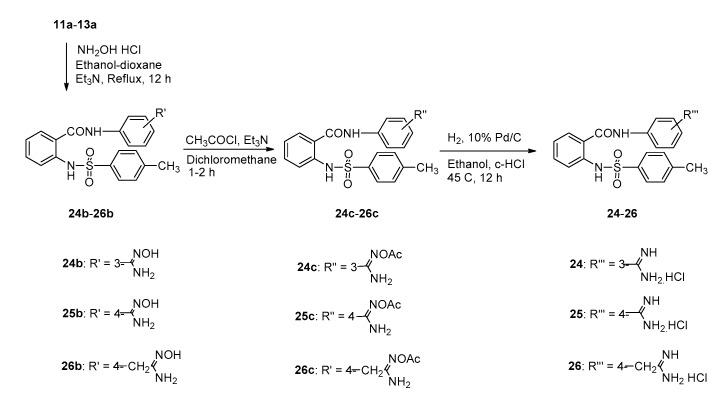
Synthetic procedures of amidino *N*^2^-tosylanthranilamide derivatives (**24**–**26**).

All synthetic methods used in this study are shown in Scheme 1, Scheme 2, Scheme 3 and Scheme 4. Scheme 1 and Scheme 2 showed the synthesis of non-amidine derivatives and Scheme 3 and Scheme 4 showed the synthesis of amidine derivatives. Scheme 1 illustrated the synthesis of *N*^2^-(thiophenecarbonyl)anthranilamide derivatives **1**–**10**, which were prepared according to synthetic procedures for *N*^3^-(thiophenecarbonyl)benzamide derivatives [22]. The non-amidine type inhibitor, rivaroxaban, apixaban, and edoxaban, possess a small lipophilic substituent, such as a methoxy or chloro or morpholine group. The 2-nitrobenzoyl chloride was coupled with 4-substituted anilines (4-chloro, 4-bromo-, 4-fluoro-, 4-methoxy-, and 4-morpholinoaniline) as small lipophilic compounds to give **1a**–**5a** and these nitro amides were reduced with Fe and NH_4_Cl to provide the corresponding amino amides **1b**–**5b**. Intermediates **1b**–**5b** were then coupled with thiophene-2-carbonyl chloride and 5-chlorothiophene-2-carbonyl chloride to furnish *N*^2^-(thiophene-2-carbonyl)anthranilamides **1**–**5** and *N*^2^-(5-chlorothiophene-2-carbonyl)anthranilamides **6**–**10**, respectively.

The introduction of sulfonamide instead of one carboxamide in the linker of diamide may give rise to differences in the possible hydrogen-binding interaction between the ligand and the residues in the thrombin/FXa active site. The *N*^2^-arylsulfonamide series **11**–**20** were synthesized to compare with the activity of *N*^2^-arylcarboxamide series **1**–**10**. *N*^2^-tosylanthranilamides were synthesized as beginning compounds for development of *N*^2^-arylsulfonamide derivatives.

The preparative route to *N*^2^-tosylanthranilamide derivatives **11**–**20** are shown in Scheme 2. Anthranilic acid was reacted with *p*-tosyl chloride to give *N*-tosylbenzoic acid, followed by chlorination with oxalyl chloride to give *N*-tosylbenzoyl chloride. This acid chloride was coupled with 3-cyano-, 4-cyano-, 4-cyanomethyl-, 4-chloro-, 4-bromo-, 4-methoxy-, 4-carboxy-, 4-(2-(2-thienyl)ethyl)-, 4-morpholino-, and 4-(2-oxomorpholino)aniline to give *N*^2^-tosylanthranilamide derivatives **11a**–**13a** and **14**–**20**, respectively. To consider the pharmacokinetic problems we replaced the amidine group with less basic residues, aminoalkyl groups. The cyano group containing compounds **11a**–**13a** were catalytically hydrogenated in the presence of 10% Pd/C and c-HCl at 45 °C to give aminoalkyl amides **11**–**13**.

Scheme 3 illustrated the synthetic procedures of amidino *N*^2^-thiophenecarbonyl anthranilamide derivatives **21**–**23**. The amidine group forms a bidentate salt bridge interaction with carboxylic acid of Asp189 in the S1 site of FXa. The high polar amidine-derived inhibitors demonstrated potent in vivo anticoagulant and antithrombotic activity. The first amide bond was obtained from acylation of 2-nitrobenzoyl chloride with 3-cyano-, 4-cyano-, and 4-cyanomethyl-aniline to afford **21a**–**23a**.

2-Aminobenzamides **21b**–**23b** were synthesized from reduction of 2-nitrobenzamides **21a**–**23a** with Fe and NH_4_Cl. The treatment of **21b**–**23b** with thiophene-2-carbonyl chloride prepared the *N*^2^-(thiophene-2-carbonyl)-*N*-(3-cyano- or -4-cyanophenyl)benzamides 21c and 22c, and the *N*^2^-(thiophene-2-carbonyl)-*N*-(4-cyanobenzyl)benzamide 23c, which were treated with hydroxylamine HCl and triethylamine to give amidoximes **21d**–**23d**. The N–O bond of amidoximes **21d**–**23d** was reduced to amidinium chloride **21**–**23** via the catalytic hydrogenation of the *O*-acetyl amidoximes **21e**–**23e** over 10% Pd/C in the presence of c-HCl at 60 psi, 45 °C for 2 h. The four proton peaks of amidine HCl salt in 21 were determined as two broad singlets at 9.08 and 9.36 ppm, and the four proton peaks of amidine HCl salt in **22** and **23** were observed as one broad singlets at 9.36 and 8.92 ppm in the ^1^H-NMR spectra, respectively. In the HMBC spectrum, two amide NH proton peaks of 22 were distinguished by observing the correlation of CO (167.5 ppm) and NH (10.77 ppm) of the -NHCO group and the correlation of CO (164.9 ppm) and NH (11.53 ppm) of the -CONH group, respectively.

Scheme 4 illustrated the synthetic procedures of amidino *N*^2^-tosylanthranilamide derivatives **24**–**26**. Compounds **11a**–**13a** were refluxed with hydroxylamine hydrochloride and triethylamine in ethanol to provide the amidoxime derivatives **24b**–**26b** to form amidinium chloride derivatives **24**–**26** via the catalytic hydrogenation of the *O*-acetyl amidoximes **24c**–**26c** in 10% Pd/C and c-HCl at 60 psi, 45 °C for 12 h.

### 2.2. Biology

#### 2.2.1. Effects of Non-Amidino *N*^2^-Aroylanthranilamides on In Vitro Activated Partial Thromboplastin Time (aPTT), Prothrombin Time (PT), In Vivo Tail Bleeding Time, and Ex Vivo Clotting Time

Many FXa and thrombin inhibitors containing an amidine group displayed insufficient absorption when administered orally, the reason is caused by strongly basic amidine group [27]. To improve oral bioavailability, synthetic projects seem to be changed to synthesis of non-amidines having weak basic groups. Rivaroxaban and apixaban are non-amidine direct FXa inhibitors, and argatroban and dabigatran eterxilate are non-amidine direct thrombin inhibitors.

The anticoagulant effects of twenty non-amidino *N*^2^-thiophenecarbonyl anthranilamides **1**–**10** and *N*^2^-tosylanthranilamides **11**–**20** were screened in aPTT and PT assays using human plasma at a concentration of 30 µg/mL in vitro and demonstrated in Table 1. As shown in Table 1, *N*-(4-morpholinophenyl)-2-(thiophen-2-ylcarbonyl)benzamide (**5**), *N*-(4-fluorophenyl)-2-(5-chloro thiophen-2-ylcarbonyl)benzamide (**8**), and *N*-(4-methoxyphenyl)-2-(5-chlorothiophen-2-ylcarbonyl)benzamide (**9**) displayed very high aPTT values (60.6, 59.3, and 65.1 s), respectively. Any *N*^2^-tosylanthranilamides **11**–**20** did not exhibit the prolongation of PT and aPTT. This result demostrate that both carboxamide linker and thiophene ring are necessary for anticoagulant activity. For the further antithrombotic experiments, thiophene compound **5** and **5**-chlorothiophene compound **9** were investigated on anticoagulant activities (in vitro and ex vivo) and on tail bleeding time (in vivo) on mouse. These two compounds were selected for further evaluations as were the ones which showed an aPPT time higher than 60 s.

According to the Table 2, aPTT in the saline-treated group was 23.6 ± 0.6 s (mean ± standard error of mean (SEM), *n* = 5) and aPTT of compounds **5**, **9**, and heparin was respectively prolonged as 33.2 ± 0.5 , 37.6 ± 0.3 and 53.3 ± 0.5 s at dose 20 µM. Although compounds **5** and **9** showed weaker activities than those of heparin, aPTT was significantly prolonged by **5** and **9** at concentrations of over 20 µM as compared to the saline-treated group. Unlike the methoxy compound **9**, the morpholine compound **5** showed PT prolongation at concentrations 20 µM and above as compared to the saline-treated group.

We can remember that a prolongation of aPTT indicates the inhibition of intrinsic pathway, and a prolongation of PT indicates the inhibition of extrinsic pathway including common pathway or not. Therefore, the prolongation of both aPTT and PT in compound **5** means inhibition of the extrinsic and intrinsic and/or common pathway. The prolongation of aPTT in **9** suggests inhibition of intrinsic pathway and/or common pathway.

The most active compounds **5** and **9** in aPTT in vitro evaluations will be evaluated in the tail bleeding times in vivo model. The circulating blood volume for mice is averagely 72 mL/kg [28]. Because the weight of used mouse is averagely 27 g, the molecular weights of **5** and **9** are 407.49 and 386.85 and the blood volume is averagely 2 mL, the amount of target compounds (24.4, 32.6, 40.8 µg/mouse for **5** and 23.2, 30.9, 38.7 µg/mouse for **9**) injected provided a maximum concentration of 30, 40, or 50 µM in the peripheral blood.

As shown in Table 3, compounds **5** and **9** significantly prolonged the tail bleeding times in at concentrations 24.4 and 23.2 µg/mouse and above as compared to the control, respectively.

The aPTT values were significantly prolonged by both **5** and **9** at concentration 24.4 and 23.2 µg/mouse and above ex vivo clotting times, while the prolongation in PT was found in compound **5**. (Table 4).

In summary, aPTT (in vitro and ex vivo) of **9** was longer than those of **5** suggesting that methoxy group of **9** is more effective for anticoagulant activity than morpholine group of **5**, while 5-chloro group of 5-chlorothiophene was seemed not to influence on the anticoagulant activity.

To obtain more active compounds, six amidino *N*^2^-aroylantranilamide derivatives (**21**–**26**) derivatives were synthesized and also screened in aPTT and PT assays using human plasma at a concentration of 30 µg/mL in vitro and were demonstrated in Table 5. In case of *N*^2^-thiophene compounds, both aPTT and PT of 3-amidino- and 4-amidinomethyl- compounds **21** and **23** were longer than 4-amidino compound **22**. In reverse, among *N*^2^-tosyl derivatives **24**–**26**, 4-amidino derivative **25** showed longer aPTT than 3-amidino- and 4-amidinomethyl- compounds **24** and **26**.

From these aPTT and PT results, **21**–**23** were further investigated on anticoagulant activities (in vitro and ex vivo) and on tail bleeding time (in vivo) on mouse. These three compounds were selected for further evaluations as were the ones which showed both aPPT and PT time higher than 50 s and 15 s, respectively.

As shown in Table 6, aPTT in the saline-treated group was 23.6 ± 0.6 s (mean ± SEM, *n* = 5) and aPTT showed 38.5 ± 0.4 s, 30.2 ± 0.3 s, 38.5 ± 0.7 s, and 53.3 ± 0.5 s at dose 20 µM in compounds **21**–**23** and heparin, respectively. aPTT of compounds **21** and **23** was significantly prolonged at concentrations of 10 µM and above, and **22** at concentrations of 20 µM and above as compared to the saline-treated group. In addition, **21**–**23** significantly showed PT prolongation (16.7 ± 0.5, 13.9 ± 0.3, 14.2 ± 0.5 s) and INR (2.04, 1.32, and 1.38) at concentrations of 20 µM and above as compared to the saline-treated group (12.4 ± 0.4 s). These results in this study showing prolongation of aPTT and PT of *N*^2^-thiophenecarbonyl anthranilamides **21**–**23** suggest inhibition of both extrinsic and intrinsic, and/or common pathway.

The most active compounds **21**–**23** in aPTT in vitro evaluations will be evaluated in the tail bleeding times in vivo model. As shown in Table 7, tail bleeding times of compounds **21** and **22** were significantly prolonged in at concentrations of 24.1 µg/mouse and above, and by compound **23** in at concentrations of 24.9 µg/mouse and above as compared to the control, respectively.

As shown Table 8, both aPTT and PT were dose-dependently prolonged by both **21** and **22** at concentration of 24.1 µg/mouse and above, and by **23** at concentrations of 24.9 µg/mouse and above ex vivo clotting times.

#### 2.2.2. Thrombin and Factor Xa (FXa) Activity

**Figure 1:**
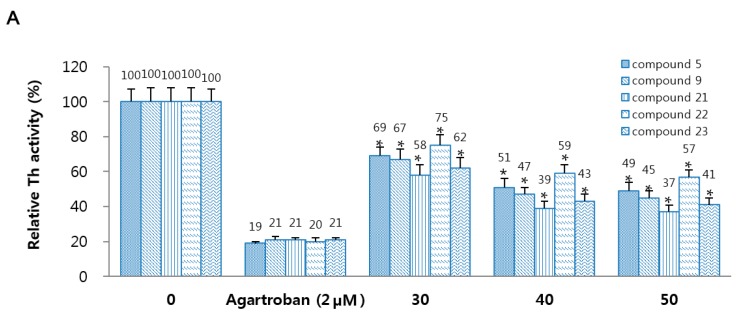

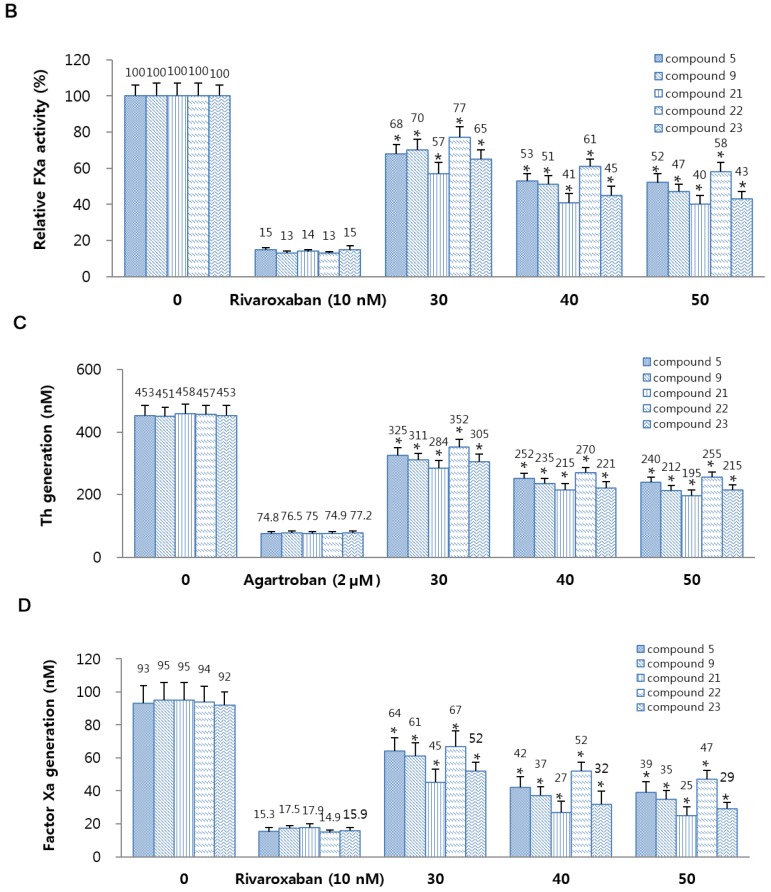
Effects of **5**, **9**, and **21**–**23** on inhibitory activity and generation of thrombin and factor Xa. (**A**) Inhibitory activity of thrombin (Th) by **5** or **9** or **21**–**23** was evaluated using a chromogenic assay. (**B**) Inhibitory activity of FXa by **5** or **9** or **21**–**23** was investigated using a chromogenic assay, as. Argatroban (**A**,**C**) or rivaroxaban (**B**,**D**) were used as positive control. (**C**) Human umbilical vein endothelial cells (HUVEC) monolayers were pre-incubated with FVa (100 pM), FXa (1 nM), and the indicated concentrations of **5** or **9** or **21**–**23** for 10 min. Prothrombin was added to a final concentration of 1 µM and prothrombin activation was determined 10 min later. (**D**) HUVECs were pre-incubated with the indicated concentrations of **5** or **9** or **21**–**23** for 10 min. After TNF-α stimulated HUVECs were incubated with FVIIa (10 nM) and FX (175 nM), FXa generation was determined. Data represent the mean ± standard error of mean (SEM) of three independent experiments. * *p* < 0.05.

To determine the fundamental mechanism of **5**, **9** and **21**–**23**, the inhibitory activities of **5**, **9** and **21**–**23** on the thrombin and FXa were investigated. According to the Figure 1A, compounds **5**, **9**, and **21**–**23** showed in a dose-dependent inhibition of the activity of thrombin. In addition, treatment with **5**, **9**, and **21**–**23** displayed in a dose-dependent inhibition of amidolytic activity of FXa, indicating direct inhibition of FXa activity. Agartroban and rivaroxaban were used as a positive control, respectively (Figure 1B).

#### 2.2.3. Thrombin and Factor Xa (FXa) Generation

According to findings reported by Sugo et al. [29], the endothelial cells are known to support the prothrombin activation by FXa. In this study, human umbilical vein endothelial cells (HUVECs) were pre-incubated with FVa and FXa in the presence of CaCl_2_ and the addition of prothrombin resulted in generation of thrombin (Figure 1C). In a previous study, Rao et al. [30] reported that the endothelium provides the functional equivalent of pro-coagulant phospholipids and supports activation of FXa, and, in TNF-α stimulated HUVECs, activated of FX by FVIIa occurred in a tissue factor (TF) expression-dependent manner. Thus, we determined the effects of **5**, **9**, and on activation of FX by FVIIa. Pre-incubation with **5**, **9**, and **21**–**23** resulted in dose-dependent inhibition of FX activation by FVIIa (Figure 1D). Therefore, these results indicated that **5**, **9**, and **21**–**23** can inhibit generation of thrombin and FXa.

#### 2.2.4. Effects of **5**, **9**, and **21**–**23** on Thrombin-Catalyzed Fibrin Polymerization and Platelet Aggregation

**Figure 2:**
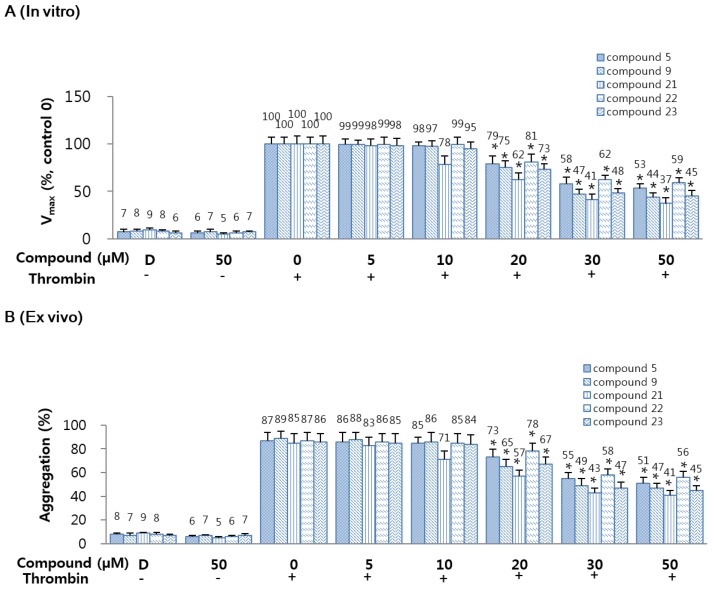

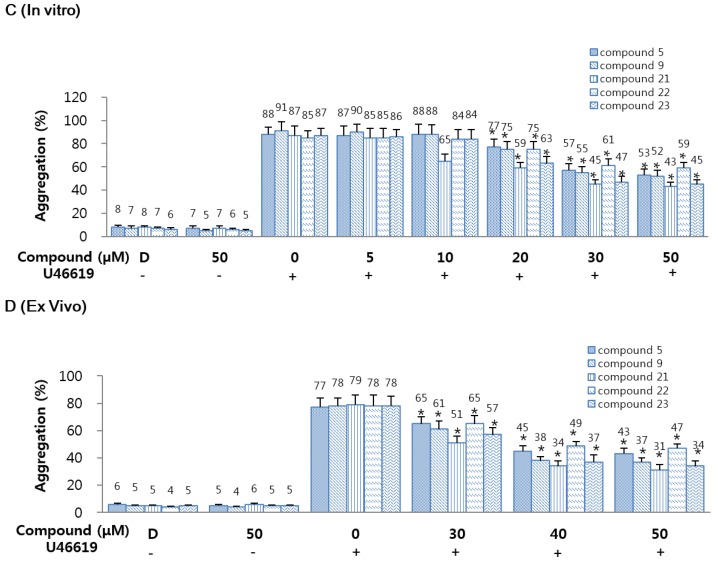
Effects of **5**, **9**, and **21**–**23** on fibrin polymerization in human plasma. (**A**) Thrombin-catalyzed fibrin polymerization at the indicated concentrations of **5** or **9** or **21**–**23** was monitored using a catalytic assay. The results are Vmax values expressed as percentages versus controls; (**B**) The effect of **5** or **9** or **21**–**23** on mouse platelet aggregation induced by 3 U/mL thrombin; (**C**) The effect of each compound on human platelet aggregation induced by 2 mM U46619; (**D**) The indicated concentration of each compound in dimethyl sulfoxide (DMSO) was injected intravenously. The effects of each compound on mouse platelet aggregation induced by 2 µM U46619 were monitored ex vivo. D: 0.2% DMSO. Data represent the mean ± SEM of three independent experiments performed. * *p* < 0.05.

The effects of **5** or **9** or **21**–**23** on thrombin-catalyzed fibrin polymerization in human plasma were monitored as changes in absorbance at 360 nm. These results display that incubation of **5** or **9** or **21**–**23** with human plasma resulted in a significant decrease in the maximal rate of fibrin polymerization (Figure 2A). To delete the effect of sample pH, all samples were diluted with 50 mM tris-buffer saline (TBS) (pH 7.4). The effect of dimethyl sulfoxide (DMSO) on human plasma was also evaluated, however, coagulation properties were unaffected. In order to exclude the possibility that the decrease of fibrin polymerization could be due to a direct effect on thrombin leading to a decrease in fibrin generation rather than polymerization of fibrin formed, reptilase-catalyzed polymerization assay was performed. Results showed that **5**, **9**, and **21**–**23** induced a significant decrease in reptilase-catalyzed polymerization [31]. To determine the antiplatelet activities of compounds **5** or **9** or **21**–**23**, thrombin-catalyzed platelet aggregation assay was performed and the results were shown in Figure 2B. Compounds **5** or **9** or **21**–**23** significantly and dose-dependently inhibited mouse platelet aggregation induced by thrombin (final concentration: 3 U/mL).

#### 2.2.5. Effects of **5**, **9**, and **21**–**23** on U46619 Catalyzed Platelet Aggregation

To determine the antiplatelet activities of **5** or **9** or **21**–**23**, a U46619 catalyzed platelet aggregation assay was carried out. As shown in Figure 2C, compounds **5** or **9** or **21**–**23** significantly and dose-dependently inhibited human platelet aggregation induced by U46619 (final concentration: 2 µM). These in vitro results were confirmed in an ex vivo platelet aggregation assay (intravenous injection, Figure 2D). As a result, **5** or **9** or **21**–**23** significantly and dose-dependently inhibited platelet aggregation induced by U46619 (final concentration: 2 µM). Until now, most of compounds containing amidine group have been known as FXa inhibitor, but non-amidines **5** and **9**, and amidines **21**–**23** exhibited the potential as platelet aggregation inhibitor.

#### 2.2.6. Cellular Viability

**Figure 3:**
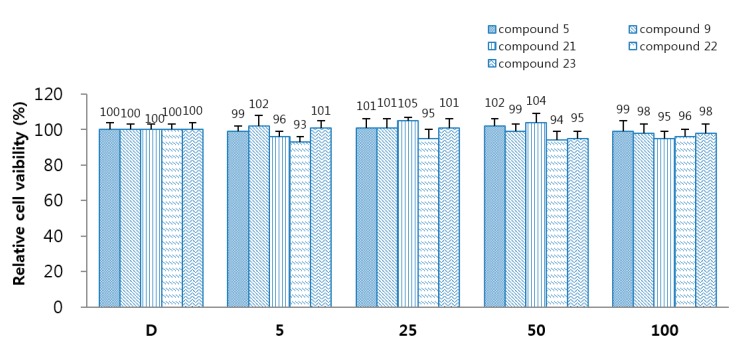
Effects of **5**, **9**, and **21**–**23** on cellular viability were determined by MTT assay. The data represent the mean ± SEM of three independent experiments performed in triplicate. D: 0.2% DMSO is the control.

To identify the cellular viability of **5**, **9**, and **21**–**23**, 3-(4,5-dimethylthiazol-2-yl)-2,5-diphenyltetrazolium bromide (MTT) assay was performed in HUVECs treated with **5**, **9**, and **21**–**23** for 24 h. Compounds **5**, **9**, and **21**–**23** did not influence cell viability at concentration up to 100 µM (Figure 3). It is safe to treat with a dose higher than the effect dose that does not show cytotoxicity even at a concentration three times higher than the effect concentration.

## 3. Materials and Methods

### 3.1. Chemistry

#### 3.1.1. Reagents and Instruments

The reagents were commercially purchased from Sigma-Aldrich (St. Louis, MO, USA) or TCI (Tokyo, Japan). If necessary, solvents were purified and/or dried prior to use. All anhydrous reactions were carried out under a dry atmosphere of nitrogen. Melting points (m.p.) were measured on Thomas-Hoover melting point apparatus (Thomas Scientific, Swedesboro, NJ, USA) and not corrected. ^1^H, ^13^C-NMR and HMBC spectra were measured on a Varian 400 MHz spectrometer (Agilent Technologies, Santa Clara, CA, USA) in DMSO-*d*_6_, CDCl_3_, or (CD_3_)_2_CO. Chemical shifts (δ) are in ppm relative to tetramethylsilane, and coupling constants (*J*) are in Hz. DIP-MS (EI) was measured on an Agilent 7890A-5975C GC/MSD (Agilent Technologies). GC/MS (EI) was determined on a SHIMADZU QP 2010 model (Shimadzu, Kyoto, Japan) and FAB-MS was determined on a JEOL JMS-700 Mstation (JEOL, Tokyo, Japan). Fraction collection was performed on an EYELA fraction collector DC-1500 (Tokyo Rikakikai, Tokyo, Japan). An analytical TLC was performed on pre-coated silica gel 60 F_254_ plates (Merck, Kenilworth, NJ, USA). Solvent systems for TLC and column chromatography were ethyl acetate/*n*-hexane mixtures and 10% methanol in dichloromethane. Column chromatography was carried out on Merck silica gel 9385 (Merck).

#### 3.1.2. General Synthetic Procedures for **1a**–**5a**

The oxalyl chloride (38.92 mmol) and triethylamine (32.93 mmol) were added to a solution of 2-nitrobenzoic acid (29.94 mmol) in anhydrous dichloromethane (50 mL) at room temperature. The reaction mixture was refluxed for 1 h and the unreacted oxalyl chloride and solvent were removed under reduced pressure and 2-nitrobenzoyl chloride was used to next reaction without purification. To a solution of 3-aminophenylcyanide or 4-aminophenylcyanide or 4-aminobenzyl cyanide (7.19 mmol) in anhydrous dichloromethane (30 mL) was added 2-nitrobenzoyl chloride (8.99 mmol) and triethylamine (7.19 mmol). The reaction mixture was stirred at room temperature for 3 h and added with water, extracted with dichloromethane (3 × 30 mL), dried with anhydrous magnesium sulfate and filtrated. The filtrate was concentrated under reduced pressure to afford the oily residue, which was separated by column chromatography to afford pure white or pale yellow compounds **1a**–**5a**.

*N*-(4′-Chlorophenyl)-2-nitrobenzamide (**1a**). Yield: 85%; m.p.: 184–186 °C (186–187 °C [32]).

*N*-(4′-Bromophenyl)-2-nitrobenzamide (**2a**) [33,34]. Yield: 86%; m.p.: 199–201 °C (202–205 °C [35]).

*N*-(4′-Fluorophenyl)-2-nitrobenzamide (**3a**). Yield: 78%; m.p.: 165–169 °C (167–171 °C [36]).

*N*-(4′-Methoxyphenyl)-2-nitrobenzamide (**4a**) [37]. Yield: 82%; m.p.: 153–154 °C.

*N*-(4′-Morpholinophenyl)-2-nitrobenzamide (**5a**). Yield: 76%; m.p.: 220–221 °C; ^1^H NMR (400 MHz, DMSO-*d*_6_) δ: 3.03 (4H, t, *J* = 4.8 Hz, H_3′′_ + H_5′′_), 3.71 (4H, t, *J* = 4.8 Hz, H_2′′_ + H_6′′_), 6.91 (2H, d, *J* = 9.0 Hz, H_3′_ + H_5′_), 7.49 (2H, d, *J* = 9.0 Hz, H_2′_ + H_6′_), 7.68–7.71 (2H, m, H_4_ + H_6_), 7.82 (1H, dt, *J* = 8.0, 1.1 Hz, H_5_), 8.09 (1H, d, *J* = 7.6 Hz, H_3_), 10.42 (1H, s, CONH); ^13^C NMR (100 MHz, DMSO-*d*_6_) δ: 48.9 (C_3′′_, C_5′′_), 66.0 (C_2′′_, C_6′′_), 115.4 (C_2′_, C_6′_), 120.8 (C_3′_, C_5′_), 124.2 (C_1_), 129.3 (C_3_), 130.8 (C_6_), 131.0 (C_1′_), 132.8 (C_4_), 134.0 (C_5_), 146.6 (C_2_), 147.8 (C_4′_), 163.4 (C=O); GC-MS (EI) *m*/*z*: 327 [M]^+^.

#### 3.1.3. General Synthetic Procedures for **1b**–**5b**

Compounds **1a**–**5a** (3.37 mmol) were dissolved in methanol (30 mL) and stirred with ammonium chloride (33.7 mmol) and iron powder (6.03 mmol) and then refluxed for 7 h. The reaction mixture was added with water and extracted with dichloromethane (3 × 40 mL), treated with anhydrous magnesium sulfate and filtrated. The filtrate was evaporated to prepare the oily residue, which was recrystallized with ethyl acetate and *n*-hexane mixture to afford pure white or pale yellow compounds **1b**–**5b**.

2-Amino-*N*-(4′-chlorophenyl)benzamide (**1b**). Yield: 63%; m.p.: 148–150 °C (147 °C [38]).

2-Amino-*N*-(4′-bromophenyl)benzamide (**2b**). Yield: 70%; m.p.: 143–145 °C (139–144 °C [39]).

2-Amino*-N*-(4′-fluorophenyl)benzamide (**3b**). Yield: 62%; m.p.: 118–120 °C (122 °C [38]).

2-Amino-*N*-(4′-methoxyphenyl)benzamide [38] (**4b**). Yield: 80%; m.p.: 118–119 °C (121 °C [38]).

2-Amino-*N*-(4′-morpholinophenyl)benzamide (**5b**). Yield: 97%; m.p.: 167–168 °C; ^1^H NMR (400 MHz, DMSO-*d*_6_ ) δ: 3.06 (4H, t, *J* = 4.8 Hz, H_3′′_ + H_5′′_), 3.74 (4H, t, *J* = 4.8 Hz, H_2′′_ + H_6′′_), 6.29 (2H, s, NH_2_), 6.57 (1H, dt, *J* = 7.7, 1.1 Hz, H_5_), 6.73 (1H, dd, *J* = 8.2, 1.0 Hz, H_3_), 6.91 (2H, d, *J* = 9.0 Hz, H_3′_ + H_5′_), 7.17 (1H, dt, *J* = 8.4, 1.5 Hz, H_4_), 7.56 (2H, d, *J* = 9.0 Hz, H_2′_ + H_6′_), 7.59 (1H, dd, *J* = 7.8, 1.8 Hz, H_6_), 9.80 (1H, s, CONH); ^13^C NMR (100 MHz, DMSO-*d*_6_) δ: 39.9 (C_3′′_, C_5′′_), 65.1 (C_2′′_, C_6′′_), 114.5 (C_1_), 115.2 (C_2′_, C_6′_), 115.6 (C_3_), 116.3 (C_5_), 121.7 (C_3′_, C_5′_), 129.5 (C_6_), 131.4 (C_1′_), 131.8 (C_4_), 147.4 (C_4′_), 149.6 (C_2_), 167.4 (C=O); GC-MS (EI) *m*/*z*: 297 [M]^+^.

#### 3.1.4. General Synthetic Procedures for **1**–**10**

Thiophene-2-carbonyl chloride and 5-chlorothiophene-2-carbonyl chloride were prepared according to the synthetic procedures for **1a**–**5a**. Compounds **1b**–**5b** (0.62 mmol) were dissolved in anhydrous benzene (30 mL) and then triethylamine (0.62 mmol) was slowly added and thiophene-2-carbonyl chloride (0.62 mmol) or 5-chlorothiophene-2-carbonyl chloride (0.62 mmol) was added. The reaction mixture was reacted at room temperature for 2 h and the following procedures were same as procedures for **1a**–**5a**. Compounds **1**–**10** were recrystallized with ethyl acetate and *n*-hexane mixture to obtain as pure white or pale yellow compounds.

*N*-(4′-Chlorophenyl)-2-(thiophen-2′′-ylcarbonylamino)benzamide (**1**).Yield: 79%; m.p.: 197–199 °C; ^1^H NMR (400 MHz, DMSO-*d*_6_) δ: 7.25 (1H, dd, *J* = 4.9, 3.8 Hz, H_4′′_), 7.29 (1H, dt, *J* = 7.7, 1.1 Hz, H_5_), 7.43 (2H, d, *J* = 8.8 Hz, H_3′_ + H_5′_), 7.61 (1H, dt, *J* = 8.0, 1.2 Hz, H_4_), 7.74–7.78 (3H, m, H_2′_ + H_6′_ + H_5′′_), 7.88–8.02 (2H, m, H_3_ + H_6_), 8.32 (1H, d, *J* = 8.0 Hz, H_3′′_), 10.63 (1H, s, NHCO), 11.54 (1H, s, CONH); ^13^C NMR (100 MHz, DMSO-*d*_6_) δ: 121.6 (C_3_), 122.5 (C_2′_, C_6′_), 123.0 (C_1_), 123.4 (C_5_), 127.9 (C_4′_), 128.4 (C_3′_, C_5′_), 128.6 (C_6_), 128.8 (C_4′′_), 129.0 (C_5′′_), 132.2 (C_4_), 132.3 (C_3′′_), 137.5 (C_2′′_), 138.1 (C_1′_), 139.5 (C_2_), 159.4 (CONH), 167.3 (NHCO); DIP-MS (EI): *m*/*z* 356 [M]^+^; HRMS (FAB): *m*/*z* calcd for C_18_H_14_ClN_2_O_2_S [M + H]^+^ 357.0465, found 357.0478.

*N*-(4′-Bromophenyl)-2-((thiophen-2′′-yl)carbonylamino)benzamide (**2**). Yield: 75%; m.p.: 210–212 °C; ^1^H NMR (400 MHz, DMSO-*d*_6_) δ: 7.25 (1H, dd, *J* = 4.9, 3.8 Hz, H_4′′_), 7.30 (1H, dt, *J* = 7.8, 1.1 Hz, H_5_), 7.56 (2H, d, *J* = 8.9 Hz, H_3′_ + H_5′_), 7.61 (1H, dt, *J* = 8.0, 1.2 Hz, H_4_), 7.71 (2H, d, *J* = 8.9 Hz, H_2′_ + H_6′_), 7.75 (1H, dd, *J* = 3.7, 1.0 Hz, H_5′′_), 7.88–8.03 (2H, m, H_3_ + H_6_), 8.30 (1H, d, *J* = 8.2 Hz, H_3′′_), 10.64 (1H, s, NHCO), 11.51 (1H, s, CONH); ^13^C NMR (100 MHz, DMSO-*d*_6_) δ: 116.0 (C_4′_), 121.6 (C_3_), 122.9 (C_2′_, C_6′_), 123.1 (C_1_), 123.4 (C_5_), 128.4 (C_6_), 128.8 (C_4′′_), 129.0 (C_5′′_), 131.5 (C_3′_, C_5′_), 132.2 (C_4_), 132.3 (C_3′′_), 138.0 (C_2′′_), 138.1 (C_1′_), 139.5 (C_2_), 159.4 (CONH), 167.3 (NHCO); DIP-MS (EI): *m*/*z* 400 [M − 1]^+^, 402 [M + 1]^+^; HRMS (FAB): *m*/*z* calcd for C_18_H_14_BrN_2_O_2_S [M + H]^+^ 400.9959, found 400.9973.

*N*-(4′-Fluorophenyl)-2-((thiophen-2′′-yl)carbonylamino)benzamide (**3**). Yield: 73%; m.p.: 215–216 °C; ^1^H NMR (400 MHz, DMSO-*d*_6_) δ: 7.19–7.26 (3H, m, H_3′_ + H_5′_+ H_4′′_), 7.29 (1H, dt, *J* = 7.7, 1.1 Hz, H_5_), 7.61 (1H, dt, *J* = 8.0, 1.0 Hz, H_4_), 7.73–7.75 (3H, m, H_2′_ + H_6′_ + H_5′′_), 7.89 (1H, dd, *J* = 5.0, 1.1 Hz, H_6_), 7.92 (1H, dd, *J* = 7.8, 1.2 Hz, H_3_), 8.36 (1H, d, *J* = 7.6 Hz, H_3′′_), 10.58 (1H, s, NHCO), 11.68 (1H, s, CONH); ^13^C NMR (100 MHz, DMSO-*d*_6_) δ: 115.9 (*J* = 22.3 Hz, C_3′_, C_5′_), 122.0 (C_3_), 123.3 (C_1_), 123.7 (*J* = 8.2 Hz, C_2′_, C_6′_), 124.1 (C_5_), 129.1 (C_6_), 129.4 (C_4′′_), 129.6 (C_5′′_), 133.0 (C_4_, C_3′′_), 135.5 (*J* = 3.0 Hz, C_1′_), 139.0 (C_2′′_), 140.2 (C_2_), 159.4 (*J* = 239.4 Hz, C_4′_), 160.0 (CONH), 167.9 (NHCO); DIP-MS (EI): *m*/*z* 340 [M]^+^; HRMS (FAB): *m*/*z* calcd for C_18_H_14_FN_2_O_2_S [M + H]^+^ 341.0760, found 341.0781.

*N*-(4′-Methoxyphenyl)-2-(thiophen-2′′-ylcarbonylamino)benzamide (**4**). Yield: 77%; m.p.: 170–172 °C; ^1^H NMR (400 MHz, DMSO-*d*_6_) δ: 3.73 (3H, s, CH_3_), 6.96 (2H, d, *J* = 9.0 Hz, H_3′_ + H_5′_), 7.31–7.23 (2H, m, H_5_ + H_4′′_), 7.59–7.64 (3H, m, H_4_ + H_2_ + H_6′_), 7.73 (1H, dd, *J* = 3.7, 0.6 Hz, H_5′′_), 7.89 (1H, dd, *J* = 5.0, 0.6 Hz, H_6_), 7.94 (1H, d, *J* = 6.9 Hz, H_3_), 8.43 (1H, d, *J* = 8.2 Hz, H_3′′_), 10.45 (1H, s, NHCO), 11.92 (1H, s, CONH); ^13^C NMR (100 MHz, DMSO-*d*_6_) δ: 55.2 (CH_3_), 113.8 (C_3′_, C_5′_), 120.9 (C_3_), 122.1 (C_1_), 122.9 (C_2′_, C_6′_), 123.2 (C_5_), 128.4 (C_6_), 128.6 (C_4′′_), 128.8 (C_5′′_), 131.3 (C_1′_), 132.2 (C_4_), 132.3 (C_3′′_), 138.5 (C_2′′_), 139.6 (C_2_), 156.1 (C_4′_), 159.3 (CONH), 167.0 (NHCO); DIP-MS (EI): *m*/*z* 352 [M]^+^; HRMS (FAB): *m*/*z* calcd for C_19_H_17_N_2_O_3_S [M + H]^+^ 353.0960, found 353.0979.

*N*-(4′-Morpholinophenyl)-2-(thiophen-2′′′-ylcarbonylamino)benzamide (**5**). Yield: 76%; m.p.: 246–247 °C; ^1^H NMR (400 MHz, DMSO-*d*_6_) δ: 3.09 (4H, t, *J* = 4.8 Hz, H_3′′_ + H_5′′_), 3.74 (4H, t, *J* = 4.8 Hz, H_2′′_ + H_6′′_), 6.96 (2H, d, *J* = 9.0 Hz, H_3′_ + H_5′_), 7.22–7.30 (2H, m, H_5_ + H_4′′′_), 7.57–7.61 (3H, m, H_4_ + H_2′_ + H_6′_), 7.72 (1H, dd, *J* = 3.7, 0.6 Hz, H_5′′′_), 7.90 (1H, dd, *J* = 4.9, 0.5 Hz, H_6_), 7.93 (1H, d, *J* = 7.3 Hz, H_3_), 8.43 (1H, d, *J* = 8.1 Hz, H_3′′′_), 10.39 (1H, s, NHCO), 11.97 (1H, s, CONH); ^13^C NMR (100 MHz, DMSO-*d*_6_) δ: 48.7 (C_3′′_, C_5′′_), 66.1 (C^2′′^, C_6′′_), 115.2 (C_3′_, C_5′_), 120.8 (C_3_), 122.0 (C_1_), 122.4 (C_2′_,C_6′_), 123.1 (C_5_), 128.4 (C_6_), 128.6 (C_4′′_), 128.8 (C_5′′_), 130.3 (C_1′_), 132.2 (C_4_), 132.3 (C_3′′_), 138.6 (C_2′′_), 139.6 (C_2_), 148.1 (C_4′_), 159.3 (CONH), 166.9 (NHCO); DIP-MS (EI): *m*/*z* 407 [M]^+^; HRMS (FAB): *m*/*z* calcd for C_22_H_22_N_3_O_3_S [M + H]^+^ 408.1382, found 408.1368.

*N*-(4′-Chlorophenyl)-2-((5-chlorothiophen-2′′-yl)carbonylamino)benzamide (**6**). Yield: 60%; m.p.: 207–209 °C; ^1^H NMR (400 MHz, DMSO-*d*_6_) δ: 7.29 (1H, d, *J* = 4.0 Hz, H_4′′_), 7.32 (1H, dt, *J* = 7.7, 1.1 Hz, H_5_), 7.43 (2H, d, *J* = 8.9 Hz, H_3′_ + H_5′_), 7.60 (1H, dt, *J* = 8.0, 1.2 Hz, H_4_), 7.64 (1H, d, *J* = 4.1 Hz, H_6_), 7.75 (2H, d, *J* = 8.9 Hz, H_2′_ + H_6′_), 7.88 (1H, dd, *J* = 7.8, 1.0 Hz, H_3_), 8.17 (1H, d, *J* = 8.2 Hz, H_3′′_), 10.63 (1H, s, NHCO), 11.44 (1H, s, CONH); ^13^C NMR (100 MHz, DMSO-*d*_6_) δ: 122.2 (C_3_), 122.4 (C_2′_, C_6′_), 123.9 (C_1_), 124.2 (C_5_), 127.8 (C_4′_), 128.6 (C_3′_, C_5′_), 128.7 (C_6_), 129.1 (C_4′′_), 132.2 (C_4_, C_3′′_), 134.2 (C_2′′_), 137.4 (C_5′′_), 137.7 (C_1′_), 138.7 (C_2_), 158.4 (CONH), 167.1 (NHCO); DIP-MS (EI): *m*/*z* 390 [M − 1]^+^; HRMS (FAB): *m*/*z* calcd for C_18_H_13_Cl_2_N_2_O_2_S [M + H]^+^ 391.0075, found 391.0089.

*N*-(4′-Bromophenyl)-2-((5-chlorothiophen-2′′-yl)carbonylamino)benzamide (**7**). Yield: 41%; m.p.: 198–199 °C; ^1^H NMR (400 MHz, DMSO-*d*_6_) δ: 7.28 (1H, d, *J* = 4.1 Hz, H_4′′_), 7.32 (1H, dt, *J* = 7.7, 1.1 Hz, H_5_), 7.55 (2H, d, *J* = 8.9 Hz, H_3′_ + H_5′_), 7.60 (1H, dt, *J* = 8.0, 1.2 Hz, H_4_), 7.64 (1H, d, *J* = 4.1 Hz, H_6_), 7.70 (2H, d, *J* = 8.9 Hz, H_2′_ + H_6′_), 7.88 (1H, dd, *J* = 7.8, 1.3 Hz, H_3_), 8.18 (1H, dd, *J* = 8.2, 0.8 Hz, H_3′′_), 10.60 (1H, s, NHCO), 11.42 (1H, s, CONH); ^13^C NMR (100 MHz, DMSO-*d*_6_) δ: 115.9 (C_4′_), 122.1 (C_3_), 122.8 (C_2′_, C_6′_), 123.9 (C_1_), 124.2 (C_5_), 128.7 (C_6_), 129.0 (C_4′′_), 131.5 (C_3′_, C_5′_), 132.1 (C_4_, C_3′′_), 134.1 (C_2′′_), 137.4 (C_5′′_), 138.0 (C_1′_), 138.6 (C_2_), 158.4 (CONH), 167.1 (NHCO); DIP-MS (EI): *m*/*z* 434 [M − 1]^+^, 436 [M + 1]^+^; HRMS (FAB): *m*/*z* calcd for C_18_H_13_BrClN_2_O_2_S [M + H]^+^ 434.9570, found 434.9549.

*N*-(4′-Fluorophenyl)-2-((5-chlorothiophen-2′′-yl)carbonylamino)benzamide (**8**). Yield: 73%; m.p.: 215–216 °C; ^1^H NMR (400 MHz, DMSO-*d*_6_) δ: 7.22 (2H, t, *J* = 8.9 Hz, H_3′_ + H_5′_), 7.27–7.34 (2H, m, H_5_ + H_4′′_), 7.64–7.57 (2H, m, H_4_ + H_6_), 7.73 (2H, dd, *J* = 9.1, 5.1 Hz, H_2′_ + H_6′_), 7.91 (1H, dd, *J* = 7.9, 1.1 Hz, H_3_), 8.24 (1H, d, *J* = 8.1 Hz, H_3′′_), 10.57 (1H, s, NHCO), 11.60 (1H, s, CONH); ^13^C NMR (100 MHz, DMSO-*d*_6_) δ: 115.2 (*J* = 22.3 Hz, C_2′_, C_6′_), 121.8 (C_3_), 123.0 (*J* = 8.1 Hz, C_3′_, C_5′_), 123.5 (C_1_), 123.8 (C_5_), 128.6 (C_6_), 128.9 (C_4′′_), 132.2 (C_4_), 134.1 (C_2′′_), 134.8 (*J* = 2.2 Hz, C_1′_), 137.7 (C_5′′_), 138.6 (C_2_), 158.3 (CONH), 158.6 (*J* = 239.5 Hz, C4′), 167.0 (NHCO); DIP-MS (EI): *m*/*z* 374 [M]^+^; HRMS (FAB): *m*/*z* calcd for C_18_H_13_ClFN_2_O_2_S [M + H]^+^ 375.0370, found 375.0388.

*N*-(4′-Methoxyphenyl)-2-((5-chlorothiophen-2′′-yl)carbonylamino)benzamide (**9**). Yield: 77%; m.p.: 190–191 °C; ^1^H NMR (400 MHz, DMSO-*d*_6_) δ: 3.75 (3H, s, CH_3_), 6.95 (2H, d, *J* = 8.8 Hz, H_3′_ + H_5′_), 7.26–7.32 (2H, m, H_5_ + H_4′′_), 7.56–7.65 (4H, m, H_4_ + H_6_ + H_2′_ + H_6′_), 7.94 (1H, d, *J* = 7.7 Hz, H_3_), 8.31 (1H, d, *J* = 8.3 Hz, H_3′′_), 10.45 (1H, s, NHCO), 11.87 (1H, s, CONH); ^13^C NMR (100 MHz, DMSO-*d*_6_) δ: 55.4 (CH_3_), 114.0 (C_2′_, C_6′_), 121.6 (C_3_), 123.0 (C_3′_, C_5′_), 123.1 (C_1_), 123.8 (C_5_), 128.7 (C_6_), 128.8 (C_4′′_), 129.1 (C_1′_), 131.5 (C_3′′_), 132.3 (C_4_), 134.4 (C_2′′_), 138.2 (C_5′′_), 139.9 (C_2_), 156.3 (C_4′_), 158.5 (CONH), 167.1 (NHCO); DIP-MS (EI): *m*/*z* 386 [M]^+^; HRMS (FAB): *m*/*z* calcd for C_19_H_16_ClN_2_O_3_S [M + H]^+^ 387.0457, found 387.0470.

*N*-(4′-Morpholinophenyl)-2-((5-chlorothiophen-2′′′-yl)carbonylamino)benzamide (**10**). Yield: 80%; m.p.: 230–231 °C; ^1^H NMR (400 MHz, DMSO-*d*_6_) δ: 3.08 (4H, t, *J* = 4.8 Hz, H_3′′_ + H_5′′_), 3.74 (4H, t, *J* = 4.8 Hz, H_2′′_ + H_6′′_), 6.96 (2H, d, *J* = 9.1 Hz, H_3′_ + H_5′_), 7.23–7.32 (2H, m, H_5_ + H_4′′′_), 7.51–7.64 (4H, m, H_4_ + H_6_ + H_2′_ + H_6′_), 7.92 (1H, dd, *J* = 7.9, 1.1 Hz, H_3_), 8.32 (1H, d, *J* = 8.3 Hz, H_3′′′_), 10.39 (1H, s, NHCO); ^13^C NMR (100 MHz, DMSO-*d*_6_) δ: 51.6 (C_3′′_, C_5′′_), 68.9 (C_2′′_, C_6′′_), 118.0 (C_3′_, C_5′_), 124.1 (C_3_), 125.2 (C_2′_, C_6′_), 125.6 (C_1_), 126.4 (C_5_), 131.2 (C_6_), 131.5 (C_4′′_), 131.7 (C_3′′_), 133.2 (C_4_), 135.0 (C_2′′_), 137.0 (C_1′_), 140.9 (C_5′′_), 141.6 (C_2_), 150.9 (C_4′_), 161.1 (CONH), 169.6 (NHCO); DIP-MS (EI): *m*/*z* 441[M]^+^; HRMS (FAB): *m*/*z* calcd for C_22_H_21_ClN_3_O_3_S [M + H]^+^ 442.0992, found 442.0977.

#### 3.1.5. General Synthetic Procedures for **11a**–**13a**

To a solution of 4-(methylphenylsulfonamido)benzoyl chloride (**2**, 2.06 mmol) in anhydrous benzene (30 mL) was added 3- or 4-aminobenzonitrile (220 mg, 1.87 mmol), 4-aminobenzylcyanide (265 mg, 1.87 mmol) and triethylamine (0.1 mL). The following procedures were same as procedures for **1a**–**5a**. Compounds **11a**–**13a** were recrystallized with dichloromethane and *n*-hexane mixture to obtain as a white powder.

*N*-(3’-Cyanophenyl)-2-(4”-methylphenylsulfonamido)benzamide (**11a**). Yield: 75%; m.p.: 216–218 °C; ^1^H NMR (400 MHz, DMSO-*d*_6_) δ: 2.23 (3H, s, CH_3_), 7.21 (2H, d, *J* = 8.4 Hz, H_3′′_ + H_5′′_), 7.23 (1H, t, *J* = 7.6 Hz, H_5_), 7.36 (1H, d, *J* = 8.0 Hz, H_3_), 7.48 (1H, t, *J* = 7.6 Hz, H_4_), 7.54–7.65 (4H, m, H_6_ + H_5′_ + H_2′′_ + H_6′′_), 7.69 (1H, d, *J* = 7.2 Hz, H_4′_), 7.88 (1H, d, *J* = 7.6 Hz, H_6′_), 8.08 (1H, s, H_2′_), 10.26 (1H, s, NHSO_2_), 10.53 (1H, s, CONH); ^13^C NMR (100 MHz, DMSO-*d*_6_) δ: 21.6 (CH_3_), 112.1 (C_3′_), 119.3 (CN), 122.9 (C_3_), 124.1 (C_1_), 125.9 (C_5_), 127.6 (C_6_), 127.8 (C_6′_), 128.3 (C_2′′_, C_6′′_), 129.8 (C_5′_), 130.4 (C_3′′_, C_5′′_), 130.8 (C_2′_), 133.1 (C_4_), 136.6 (C_4′_), 137.1 (C_1′′_), 137.4 (C_4′′_), 140.0 (C_1′_), 144.3 (C_2_), 167.5 (C=O); DIP-MS (EI): *m*/*z* 391 [M]^+^.

*N*-(4′-Cyanophenyl)-2-(4”-methylphenylsulfonamido)benzamide (**12a**). Yield: 70%; m.p.: 253–255 °C; ^1^H NMR (400 MHz, DMSO-*d*_6_) δ: 2.22 (3H, s, CH_3_), 7.12–7.26 (3H, m, H_5_ + H_3′′_ + H_5′′_), 7.33 (1H, d, *J* = 7.6 Hz, H_3_), 7.47 (1H, t, *J* = 7.6 Hz, H_4_), 7.56 (2H, d, *J* = 7.6 Hz, H_2′′_ + H_6′′_), 7.67 (1H, d, *J* = 7.2 Hz, H_6_), 7.77–7.87 (4H, m, H_2′_ + H_3′_ + H_5′_ + H_6′_), 10.16 (1H, s, NHSO_2_), 10.59 (1H, s, CONH); ^13^C NMR (100 MHz, DMSO-*d*_6_) δ: 21.6 (CH_3_), 106.4 (C_4′_), 119.7 (CN), 121.2 (C_2′_, C_6′_), 123.1 (C_3_), 125.2 (C_1_), 126.4 (C_5_), 127.5 (C_6_), 128.0 (C_2′′_, C_6′′_), 130.4 (C_3′′_, C_5′′_), 133.1 (C_3′_, C_5′_), 133.8 (C_4_), 137.2 (C_1′′_, C_4′′_), 143.6 (C_1′_), 144.3 (C_2_), 167.6 (C=O); DIP-MS (EI): *m*/*z* 391 [M]^+^.

*N*-(4′-Cyanomethylphenyl)-2-(4”-methylphenylsulfonamido)benzamide (**13a**). Yield: 68%; m.p.: 142–146 °C; ^1^H NMR (400 MHz, DMSO-*d*_6_) δ: 2.27 (3H, s, CH_3_), 4.02 (2H, s, CH_2_), 7.20–7.29 (3H, m, H_5_ + H_3′′_ + H_5′′_), 7.36 (2H, d, *J* = 8.8 Hz, H_3′_ + H_5′_), 7.45–7.53 (2H, m, H_3_ + H_4_), 7.67 (2H, d, *J* = 8.4 Hz, H_2′′_ + H_6′′_), 7.76 (1H, dd, *J* = 8.0, 1.2 Hz, H_6_), 10.39 (1H, s, NHSO_2_), 10.59 (1H, s, CONH); ^13^C NMR (100 MHz, DMSO-*d*_6_) δ: 21.0 (CH_3_), 21.9 (CH_2_), 119.3 (CN), 121.4 (C_2′_, C_6′_), 124.0 (C_3_), 124.1 (C_1_), 126.8 (C_5_), 127.0 (C_6_), 128.4 (C_2′′_, C_6′′_), 129.1 (C_3′_, C_5′_), 129.8 (C_3′′_, C_5′′_), 132.4 (C_4_), 135.9 (C_1′_), 137.2 (C_1′′_), 137.7 (C_4′′_), 143.7 (C_2_), 166.7 (C=O); GC-MS (EI) *m*/*z*: 405 [M]^+^.

#### 3.1.6. General Synthetic Procedures for **11**–**13**

To a suspension of 10% Pd-C (200 mg) in ethanol (50 mL) was added 1*N*-HCl (1 mL) and nitriles **11a**–**13a** (1.28 mmol) and the reaction mixture was catalytically hydrogenated in parr hydrogenation apparatus (50 psi of hydrogen) at room temperature for 6 h. When the starting material could be no longer detected by TLC, the reaction mixture was filtrated on celite pad. The filtrate was concentrated to give the oily product which was diluted in ethanol and then crystallized with diethyl ether to form the light brown precipitate which was recrystallized with ethanol and diethyl ether mixture to afford **11**–**13** as a white powder.

*N*-(3’-Aminomethyl)phenyl-2-(4”-methylphenylsulfonamido)benzamide (**11**). Yield: 72%; m.p: 163–166 °C; ^1^H NMR (400 MHz, CD_3_OD) δ: 2.29 (3H, s, CH_3_), 4.03 (2H, s, CH_2_), 7.23 (1H, t, *J* = 7.2 Hz, H_4_), 7.27 (2H, d, *J* = 8.4 Hz, H_3′′_ + H_5′′_), 7.32 (1H, d, *J* = 7.6 Hz, H_4′_), 7.40–7.45 (2H, m, H_5′_ + H_6′_), 7.42 (1H, t, *J* = 7.6 Hz, H_5_), 7.55 (1H, d, *J* = 8.4 Hz, H_3_), 7.63 (2H, d, *J* = 8.4 Hz, H_2′′_ + H_6′′_), 7.83 (1H, d, *J* = 7.6 Hz, H_6_), 7.90 (1H, s, H_2′_), 8.50 (3H, br s, aminium chloride), 10.52 (1H, s, NHSO_2_), 10.69 (1H, s, CONH); ^13^C NMR (100 MHz, DMSO-*d*_6_) δ: 20.9 (CH_3_), 42.3 (CH_2_), 121.0 (C_2′_), 121.2 (C_6′_), 121.5 (C_3_), 123.6 (C_1_), 124.8 (C_5_), 126.8 (C_2′′_, C_6′′_), 128.9 (C_6_), 129.2 (C_4′_), 129.8 (C_3′′_, C_5′′_), 132.5 (C_4_, C_1′_), 134.5 (C_3′_), 137.4 (C_1′′_), 138.5 (C_4′′_), 143.7 (C_2_), 166.8 (C=O); DIP-MS (EI): *m*/*z* 395 [M − HCl]^+^; HRMS (FAB): calcd for C_21_H_22_N_3_O_3_S [M + H]^+^ 396.1382, found 396.1396.

*N*-(4′-Aminomethyl)phenyl-2-(4”-methylphenylsulfonamido)benzamide (**12**). Yield: 81%; m.p: 92–95 °C; ^1^H NMR (400 MHz, CD_3_OD) δ: 2.29 (3H, s, CH_3_), 4.03 (2H, s, CH_2_), 7.18–7.24 (3H, m, H_4_ + H_3′′_ + H_5′′_), 7.42 (1H, t, *J* = 8.0 Hz, H_5_), 7.45–7.53 (3H, m, H_3_ + H_3′_+ H_5′_), 7.61 (2H, d, *J* = 8.4 Hz, H_2′′_ + H_6′′_), 7.69 (2H, d, *J* = 8.4 Hz, H_2′_ + H_6′_), 7.80 (1H, d, *J* = 8.0 Hz, H_6_), 8.36 (3H, br s, aminium chloride), 10.44 (1H, s, NHSO_2_), 10.61 (1H, s, CONH); ^13^C NMR (100 MHz, DMSO-*d*_6_) δ: 20.9 (CH_3_), 41.8 (CH_2_), 120.9 (C_2′_, C_6′_), 121.4 (C_3_), 124.0 (C_1_), 124.1 (C_5_), 126.8 (C_2′′_, C_6′′_), 129.1 (C_6_), 129.3 (C_3′_, C_5′_), 129.8 (C_3′′_, C_5′′_), 132.4 (C_4_, C_1′_), 135.9 (C_4′_), 137.2 (C_1′′_), 138.4 (C_4′′_), 143.7 (C_2_), 166.7 (C=O); DIP-MS (EI): *m*/*z* 395 [M − HCl]^+^; HRMS (FAB): calcd for C_21_H_22_N_3_O_3_S [M]^+^ 396.1382, found 396.1399.

*N*-(4′-Aminoethyl)phenyl-2-(4”-methylphenylsulfonamido)benzamide (**13**). Yield: 77%; m.p: 253–255 °C; ^1^H NMR (400 MHz, CD_3_OD) δ: 2.27 (3H, s, CH_3_), 2.98 (2H, t, *J* = 7.8 Hz, CH_2_), 3.21 (2H, t, *J* = 7.8 Hz, CH_2_), 7.14 (2H, m, H_3′_ + H_5′_), 7.23 (1H, t, *J* = 7.6 Hz, H_4_), 7.30 (2H, d, *J* = 8.0 Hz, H_3′′_ + H_5′′_), 7.46–7.61 (6H, m, H_3_ + H_5_ + H_2′_ + H_6′_ + H_2′′_ + H_6′′_), 7.67 (1H, d, *J* = 7.6 Hz, H_6_), 8.33 (3H, br s, aminium chloride), 10.41 (1H, s, NHSO_2_), 10.63 (1H, s, CONH); ^13^C NMR (100 MHz, DMSO-*d*_6_) δ: 20.2 (CH_3_), 32.9 (CH_2_), 40.7 (CH_2_), 121.8 (C_2′_, C_6′_), 123.5 (C_3_), 124.8 (C_1_), 125.3 (C_5_), 127.1 (C_2′′_, C_6′′_), 128.2 (C_6_), 129.0 (C_3′_, C_5′_), 129.5 (C_3′′_, C_5′′_), 132.2 (C_4_), 133.2 (C_4′_), 136.1 (C_1′_), 137.3 (C_1′′_), 137.7 (C_4′′_), 144.1 (C_2_), 167.7 (C=O); DIP-MS (EI): *m*/*z* 409 [M − HCl]^+^; HRMS (FAB): calcd for C_22_H_24_N_3_O_3_S [M + H]^+^ 410.1538, found 410.1520.

#### 3.1.7. General Synthetic Procedures for **14**–**20**

To a solution of 4-(methylphenylsulfonamido)benzoyl chloride (**2**, 1.50 mmol) was dissolved in anhydrous benzene (30 mL) was added 4-chloroaniline or 4-bromoaniline or 4-methoxyaniline or 4-carboxyaniline or 2-(thiophen-2-yl)ethylamine or 4-morpholinoaniline or 4-(2-oxomorpholino)aniline (1.36 mmol) and triethylamine (0.1 mL). The following procedures were same as procedures for **1a**–**5a**. Compounds **14**–**20** were recrystallized with dichloromethane and *n*-hexane mixture to afford a white powder, respectively.

*N*-(4′-Chlorophenyl)-2-(4"-methylphenylsulfonamido)benzamide (**14**). Yield: 82%; m.p: 220–223 °C; ^1^H NMR (400 MHz, DMSO-*d*_6_) δ: 2.29 (3H, s, CH_3_), 7.20–7.30 (3H, m, H_4_ + H_3′′_ + H_5′′_), 7.58 (2H, d, *J* = 7.6 Hz, H_3′_ + H_5′_), 7.69 (2H, d, *J* = 8.0 Hz, H_2′′_ + H_6′′_), 7.71 (2H, d, *J* = 7.6 Hz, H_2′_ + H_6′_), 7.98 (1H, t, *J* = 7.6 Hz, H_5_), 8.03 (1H, d, *J* = 8.0 Hz, H_3_), 8.18 (1H, d, *J* = 8.0 Hz, H_6_) 11.95 (1H, s, CONH); ^13^C NMR (100 MHz, DMSO-*d*_6_) δ: 21.6 (CH_3_), 121.2 (C_2′_, C_6′_), 123.1 (C_3_), 125.2 (C_1_), 126.4 (C_5_), 127.5 (C_6_), 128.0 (C_2′′_, C_6′′_), 129.9 (C_4′_), 130.4 (C_3′′_, C_5′′_), 133.1 (C_4_, C_3′_, C_5′_), 136.8 (C_1′′_), 137.2 (C_4′′_), 144.3 (C_2_), 167.6 (C=O); DIP-MS (EI) *m*/*z*: 400 [M]^+^; HRMS (FAB): calcd for C_20_H_18_ClN_2_O_3_S [M + H]^+^ 401.0727, found 401.0741.

*N*-(4′-Bromophenyl)-2-(4"-methylphenylsulfonamido)benzamide (**15**). Yield: 80%; m.p: 211–213 °C; ^1^H NMR (400 MHz, CDCl_3_) δ: 2.26 (3H, s, CH_3_), 7.12–7.21 (3H, m, H_4_ + H_3′′_ + H_5′′_), 7.38 (2H, d, *J* = 8.8 Hz, H_3′_ + H_5′_), 7.43–7.51 (4H, m, H_3_ + H_5_ + H_2′_ + H_6′_), 7.62 (2H, d, *J* = 8.4 Hz, H_2′′_ + H_6′′_), 7.69 (1H, d, *J* = 7.8 Hz, H_6_) 10.1 (1H, s, NHSO_2_), 10.45 (1H, s, CONH); ^13^C NMR (100 MHz, CDCl_3_) δ: 21.7 (CH_3_), 118.2 (C_4′_), 122.2 (C_2′_, C_6′_), 122.7 (C_3_), 122.9 (C_1_), 124.3 (C_5_), 126.9 (C_6_), 127.5 (C_2′′_, C_6′′_), 129.8 (C_3′′_, C_5′′_), 132.3 (C_3′_, C_5′_), 133.3 (C_4_), 136.4 (C_1′_), 136.5 (C_1′′_), 138.8 (C_4′′_), 144.0 (C_2_), 166.7 (C=O); DIP-MS (EI) *m*/*z*: 444 [M]^+^; HRMS (FAB): calcd for C_20_H_18_BrN_2_O_3_S [M + H]^+^ 445.0222, found 445.0239.

*N*-(4′-Methoxyphenyl)-2-(4"-methylphenylsulfonamido)benzamide (**16**). Yield: 87%; m.p: 157–159 °C; ^1^H NMR (400 MHz, CDCl_3_) δ: 2.28 (3H, s, CH_3_), 3.76 (3H, s, OCH_3_), 6.95 (2H, d, *J* = 8.8 Hz, H_3′_ + H_5′_), 7.21 (1H, t, *J* = 6.8 Hz, H_4_), 7.26 (2H, d, *J* = 8.0 Hz, H_3′′_ + H_5′′_), 7.44–7.50 (2H, m, H_3_ + H_5_), 7.54 (2H, d, *J* = 8.8 Hz, H_2′_+ H_6′_), 7.61 (2H, d, *J* = 8.4 Hz, H_2′′_ + H_6′′_), 7.77 (1H, d, *J* = 7.6 Hz, H_6_), 10.23 (1H, s, NHSO_2_), 10.81 (1H, s, CONH); ^13^C NMR (100 MHz, CDCl_3_) δ: 21.6 (CH_3_), 55.9 (OCH_3_), 114.4 (C_3′_, C_5′_), 121.6 (C_3_), 123.4 (C_2′_, C_6′_), 124.0 (C_1_), 124.6 (C_5_), 127.5 (C_2′′_, C_6′′_), 129.6 (C_6_), 130.5 (C_3′′_, C_5′′_), 131.8 (C_1′_), 133.0 (C_4_), 136.5 (C_1′′_), 138.2 (C_4′′_), 144.4 (C_2_), 156.8 (C_4′_), 167.0 (C=O) DIP-MS (EI) *m*/*z*: 396 [M]^+^; HRMS (FAB): calcd for C_21_H_21_N_2_O_4_S [M + H]^+^ 397.1222, found 397.1241.

*N*-(4′-Carboxyphenyl)-2-(4"-methylphenylsulfonamido)benzamide (**17**). Yield: 85%; m.p: 210–212 °C; ^1^H NMR (400 MHz, DMSO-*d*_6_) δ: 2.23 (3H, s, CH_3_), 7.18–7.25 (3H, m, H_4_ + H_3′′_ + H_5′′_), 7.36 (1H, d, *J* = 8.4 Hz, H_3_), 7.47 (1H, t, *J* = 7.6 Hz, H_5_), 7.57 (2H, d, *J* = 8.0 Hz, H_2′′_ + H_6′′_), 7.61 (2H, d, *J* = 8.8 Hz, H_2′_ + H_6′_), 7.86 (2H, d, *J* = 8.8 Hz, H_3′_ + H_5′_), 7.71 (1H, d, *J* = 8.0 Hz, H_6_), 10.31 (1H, s, NHSO_2_), 10.52 (1H, s, CONH); ^13^C NMR (100 MHz, DMSO-*d*_6_) δ: 21.3 (CH_3_), 117.5 (C_2′_, C_6′_), 122.0 (C_3_), 124.3 (C_1_), 124.3 (C_5_), 126.8 (C_4′_), 127.1 (C_2′′_, C_6′′_), 129.0 (C_6_), 128.9 (C_3′′_, C_5′′_), 130.8 (C_3′_, C_5′_*)*, 132.8 (C_4_), 136.1 (C_1′′_), 138.5 (C_4′′_), 144.1 (C_2_), 167.3 (C=O) DIP-MS (EI) *m*/*z*: 410 [M]^+^; HRMS (FAB): calcd for C_21_H_19_N_2_O_5_S [M + H]^+^ 411.1015, found 411.1036.

*N*-(2-(thiophen-2′-yl)ethyl)-2-(4"-methylphenylsulfonamido)benzamide (**18**). Yield: 60%; m.p: 119–121 °C; ^1^H NMR (400 MHz, CDCl_3_) δ: 2.31 (3H, s, CH_3_), 3.02 (2H, t, *J* = 6.8 Hz, CH_2_), 3.45 (2H, q, *J* = 6.8 Hz, CH_2_), 6.91 (1H, d, *J* = 2.4 Hz, H_3′_), 6.96 (1H, d, *J* = 5.2, 3.6 Hz, H_4′_), 7.13 (1H, t, *J* = 7.2 Hz, H_4_), 7.31 (2H, d, *J* = 8.0 Hz, H_3′′_ + H_5′′_), 7.35 (1H, dd, *J* = 5.2, 1,2 Hz, H_5′_), 7.42–7.53 (2H, m, H_3_ + H_5_), 7.61 (2H, d, *J* = 8.4 Hz, H_2′′_ + H_6′′_), 7.66 (1H, d, *J* = 8.0 Hz, H_6_), 8.91 (1H, s, NHSO_2_), 11.57 (1H, s, CONH); ^13^C NMR (100 MHz, CDCl_3_) δ: 21.0 (CH_3_), 28.7 (CH_2_), 40.9 (CH_2_), 119.7 (C_3_), 120.6 (C_1_), 123.4 (C_5_), 124.2 (C_5′_), 125.3 (C_4′_), 126.8 (C_2′′_, C_6′′_), 127.0 (C_3′_), 128.3 (C_6_), 129.9 (C_3′′_, C_5′′_), 132.4 (C_4_), 135.9 (C_1′′_), 138.4 (C_4′′_), 141.2 (C_2′_), 143.8 (C_2_), 168.0 (C=O); DIP-MS (EI) *m*/*z*: 400 [M]^+^; HRMS (FAB): calcd for C_20_H_21_N_2_O_3_S_2_ [M + H]^+^ 401.0994, found 401.0982.

*N*-(4′-morpholinophenyl)-2-(4"-methylphenylsulfonamido)benzamide (**19**). Yield: 55%; m.p: 206–209 °C; ^1^H NMR (400 MHz, CDCl_3_) δ: 2.29 (3H, s, CH_3_), 3.09 (4H, t, *J* = 4.8 Hz, CH_2_), 3.75 (4H, t, *J* = 4.4 Hz, CH_2_), 6.95 (2H, d, *J* = 8.8 Hz, H_3′_ + H_5′_), 7.20 (1H, t, *J* = 6.0 Hz, H_4_), 7.26 (2H, d, *J* = 8.4 Hz, H_3′′_ + H_5′′_), 7.45–7.53 (4H, m H_3_ + H_5_ + H_2′_ *+* H_6′_), 7.61 (2H, d, *J* = 8.4 Hz, H_2′′_ + H_6′′_), 7.77 (1H, d, *J* = 7.6 Hz, H_6_), 10.18 (1H, s, NHSO_2_), 10.89 (1H, s, CONH); ^13^C NMR (100 MHz, CDCl_3_) δ: 21.7 (CH_3_), 49.4 (2 x CH_2_), 66.8 (2 x CH_2_), 115.8 (C_3′_, C_5′_), 122.9 (C_3_), 123.9 (C_1_, C_2′_, C_6′_), 124.6 (C_5_), 127.6 (C_2′′_, C_6′′_), 129.6 (C_6_), 130.5 (C_3′′_, C_5′′_), 130.7 (C_1′_), 133.0 (C_4_), 136.5 (C_1′′_), 138.2 (C_4′′_), 144.4 (C_2_), 148.8 (C_4′_), 166.9 (C=O); DIP-MS (EI) *m*/*z*: 451 [M]^+^; HRMS (FAB): calcd for C_24_H_26_N_3_O_4_S [M + H]^+^ 452.1644, found 452.1627.

*N*-(4′-(2-oxomorpholino)phenyl)-2-(4"-methylphenylsulfonamido)benzamide (**20**). Yield: 44%; m.p: 270–273 °C; ^1^H NMR (400 MHz, CDCl_3_) δ: 2.28 (3H, s, CH_3_), 3.74 (2H, t, *J* = 4.8 Hz, CH_2_), 3.99 (2H, t, *J* = 4.8 Hz, CH_2_), 4.21 (2H, s, CH_2_), 7.20–7.30 (3H, m, H_4_ + H_3′_ + H_5′_), 7.39 (2H, d, *J* = 8.8 Hz, H_3′′_ + H_5′′_), 7.40–7.52 (2H, m, H_3_ + H_5_), 7.61 (2H, d, *J* = 8.4 Hz, H_2′_+ H_6′_), 7.67 (2H, d, *J* = 8.8 Hz, H_2′′_ + H_6′′_), 7.77 (1H, d, *J* = 7.6 Hz, H_6_), 10.40 (1H, s, NHSO_2_), 10.61 (1H, s, CONH); ^13^C NMR (100 MHz, CDCl_3_) δ: 21.0 (CH_3_), 40.1 (CH_2_), 63.5 (CH_2_), 67.8 (CH_2_), 117.8 (C_3′_, C_5′_), 121.2 (C_3_), 124.0 (C_1_), 124.1 (C_5_), 125.7 (C_2′_, C_6′_), 126.8 (C_2′′_, C_6′′_), 129.1 (C_6_), 129.8 (C_3′′_, C_5′′_), 132.4 (C_4_), 135.9 (C_1′_), 136.4 (C_4′_), 137.2 (C_1′′_), 137.7 (C_4′′_), 143.8 (C_2_), 166.0 (C=O), 166.6 (C=O); DIP-MS (EI) *m*/*z*: 465 [M]^+^; HRMS (FAB): calcd for C_24_H_24_N_3_O_5_S [M + H]^+^ 466.1437, found 466.1454.

#### 3.1.8. General Synthetic Procedures for **21a**–**23a**

2-Nitrobenzoyl chloride (8.99 mmol) and triethylamine (7.19 mmol) were added to a solution of 3-aminophenylcyanide or 4-aminophenylcyanide or 4-aminobenzyl cyanide (7.19 mmol) in anhydrous dichloromethane (30 mL) and stirred at room temperature for 3 h. The reaction was terminated with water and extracted with dichloromethane (3 × 30 mL), dried with anhydrous magnesium sulfate and filtrated. The filtrate was concentrated to give the crude oily reside, which was recrystallized with ethyl acetate and *n*-hexane mixture to afford pure white or pale yellow compounds **21a**–**23a**.

*N*-(3′-Cyanophenyl)-2-nitrobenzamide (**21a**). Yield: 80%; m.p.: 170–171 °C; ^1^H NMR (400 MHz, DMSO-*d*_6_) δ: 7.59–7.92 (6H, m, H_4_ + H_5_ + H_6_ + H_4′_ + H_5′_ + H_6′_), 8.13 (1H, s, H_2′_), 8.18 (1H, d, *J* = 8.0 Hz, H_3_), 11.02 (1H, s, CONH); ^13^C NMR (100 MHz, DMSO-*d*_6_) δ: 111.7 (C_3′_), 118.6 (C≡N), 122.2 (C_6′_), 124.2 (C_5′_), 124.4 (C_1_), 127.5 (C_2′_), 129.3 (C_3_), 130.4 (C_4′_), 131.3 (C_6_), 132.0 (C_4_), 134.3 (C_5_), 139.6 (C_1′_), 146.3 (C_2_), 164.6 (C=O); GC-MS (EI) *m*/*z*: 267 [M]^+^.

*N*-(4′-Cyanophenyl)-2-nitrobenzamide (**22a**). Yield: 84%; m.p.: 217–218 °C; ; ^1^H NMR (400 MHz, DMSO-*d*_6_) δ: 7.77–7.84 (6H, m, H_4_ + H_6_ + H_2′_ + H_3′_ + H_5′_ + H_6′_), 7.90 (1H, td, *J* = 7.6, 1.2 Hz, H_5_), 8.19 (1H, dd, *J* = 8.0, 4.0 Hz, H_3_), 11.08 (1H, s, CONH); ^13^C NMR (100 MHz, DMSO-*d*_6_) δ: 105.7 (C_4′_), 118.9 (C≡N), 119.6 (C_2′_, C_6′_), 124.8 (C_1_), 129.3 (C_3_), 131.3 (C_6_), 132.1 (C_4_), 133.4 (C_3′_, C_5′_), 134.3 (C_5_), 142.9 (C_1′_), 146.2 (C_2_), 164.7 (C=O); GC-MS (EI) *m*/*z*: 267 [M]^+^.

*N*-(4′-Cyanomethylphenyl)-2-nitrobenzamide (**23a**). Yield: 76%; m.p.: 174–175 °C; ^1^H NMR (400 MHz, DMSO-*d*_6_) δ: 4.01 (2H, s, CH_2_), 7.35 (2H, d, *J* = 8.0 Hz, H_3′_ + H_5′_), 7.68 (2H, d, *J* = 8.0 Hz, H_2′_ + H_6′_), 7.75–7.79 (2H, m, H_4_ + H_6_), 7.88 (1H, t, *J* = 8.0 Hz, H_5_), 8.16 (1H, d, *J* = 8.0 Hz, H_3_), 10.71 (1H, s, CONH); ^13^C NMR (100 MHz, DMSO-*d*_6_) δ: 21.9 (CH_2_), 119.3 (C≡N), 120.0 (C_2′_, C_6′_), 124.3 (C_1_), 126.6 (C_4′_), 128.6 (C_3′_, C_5′_), 129.3 (C_3_), 131.0 (C_6_), 132.5 (C_4_), 134.1 (C_5_), 138.2 (C_1′_), 146.4 (C_2_), 164.1 (C=O); GC-MS (EI) *m*/*z*: 281 [M]^+^.

#### 3.1.9. General Synthetic Procedures for **21b**–**23b**

To a nitro compounds **21a**–**23a** (1.79 mmol) in methanol (20 mL) was added ammonium chloride (17.9 mmol) and iron powder (3.58 mmol), and then the reaction mixture was refluxed for 7 h. The following procedures were same as synthetic procedures for **1b**–**5b**. Compounds **21b**–**23b** were recrystallized with ethyl acetate and *n*-hexane mixture to provide pure white or pale-yellow compounds **21b**–**23b**.

2-Amino-*N*-(3′-cyanophenyl)benzamide (**21b**). Yield: 82%; m.p.: 158–160 °C, (161–163 °C [40]).

2-Amino-*N*-(4′-cyanophenyl)benzamide (**22b**). Yield: 64%; m.p.: 233–234 °C, (231 °C [40]).

2-Amino-*N*-(4′-cyanomethylphenyl)benzamide (**23b**). Yield: 61%; m.p.: 144–145 °C; ^1^H NMR (400 MHz, DMSO-*d*_6_) δ: 3.98 (2H, s, CH_2_), 6.32 (2H, s, NH_2_), 6.59 (1H, t, *J* = 6.8 Hz, H_5_), 6.75 (1H, d, *J* = 7.6 Hz, H_3_), 7.18–7.22 (2H, m, H_4_ + H_6_), 7.30 (2H, d, *J* = 8.0 Hz, H_3′_ + H_5′_), 7.73 (2H, d, *J* = 8.0 Hz, H_2′_ + H_6′_), 10.03 (1H, s, CONH); ^13^C NMR (100 MHz, DMSO-*d*_6_) δ: 21.8 (CH_2_), 114.6 (C_1_), 115.0 (C_3_), 116.3 (C_5_), 119.3 (C≡N), 120.8 (C_2′_, C_6′_), 128.2 (C_3′_, C_5′_), 128.6 (C_4′_), 129.3 (C_6_), 132.1 (C_4_), 138.6 (C_1′_), 149.7 (C_2_), 167.8 (C=O); DIP-MS (EI) *m*/*z*: 251 [M]^+^.

#### 3.1.10. General Synthetic Procedures of **21c**–**23c**

Compounds **21b**–**23b** (1.48 mmol) were dissolved in anhydrous benzene (20 mL) and added with triethylamine (1.48 mmol). To the stirred solution, thiophene-2-carbonyl chloride (1.85 mmol) was added at room temperature and then refluxed for 1 h. The following procedures were same as synthetic procedures for **1**–1**0**. Compounds **21c**–**23c** were recrystallized with ethyl acetate and *n*-hexane mixture to give pure white or pale yellow compounds **21c**–**23c**.

*N*-(3′-Cyanophenyl)-2-((thiophen-2′′-yl)carbonylamino)benzamide (**21c**). Yield: 98%; m.p.: 208–209 °C; ^1^H NMR (400 MHz, DMSO-*d*_6_) δ: 7.25 (1H, dd, *J* = 5.0, 4.0 Hz, H_4′′_), 7.32 (1H, td, *J* = 7.6, 1.2 Hz, H_5_), 7.56–7.65 (3H, m, H_4_ + H_4′_ + H_6′_), 7.80 (1H, dd, *J* = 3.8, 0.8 Hz, H_5′′_), 7.88–7.91 (2H, m, H_3_ + H_6_), 7.99–8.02 (1H, m, H_5′_), 8.19 (1H, s, H_2′_), 8.24 (1H, dd, *J* = 8.2, 0.8 Hz, H_3′′_), 10.80 (1H, s, NHCO), 11.36 (1H, s, CONH); ^13^C NMR (100 MHz, DMSO-*d*_6_) δ: 111.2 (C_3′_), 118.5 (C≡N), 121.8 (C_3_), 123.3 (C_1_), 124.5 (C_5_), 125.2 (C_6′_), 127.3 (C_2′_), 128.2 (C_6_), 128.7 (C_4′′_), 128.8 (C_5′′_), 129.9 (C_4′_), 132.0 (C_4_), 132.1 (C_3′′_), 137.6 (C_2′′_), 139.2 (C_1′_), 139.3 (C_2_), 159.5 (CONH), 167.5 (NHCO); DIP-MS (EI) *m*/*z*: 347 [M]^+^.

*N*-(4′-Cyanophenyl)-2-((thiophen-2′′-yl)carbonylamino)benzamide (**22c**). Yield: 69%; m.p.: 266–267 °C; ^1^H NMR (400 MHz, DMSO-*d*_6_) δ: 7.24 (1H, t, *J* = 4.0 Hz, H_4′′_), 7.32 (1H, t, *J* = 7.6 Hz, H_5_), 7.62 (1H, t, *J* = 8.0 Hz, H_4_), 7.79 (1H, d, *J* = 3.2 Hz, H_5′′_), 7.83 (2H, d, *J* = 8.0 Hz, H_3′_ + H_5′_), 7.87–7.89 (2H, m, H_3_ + H_6_), 7.94 (2H, d, *J* = 8.0 Hz, H_2′_ + H_6′_), 8.20 (1H, d, *J* = 8.0 Hz, H_3′′_), 10.86 (1H, s, NHCO), 11.23 (1H, s, CONH); ^13^C NMR (100 MHz, DMSO-*d*_6_) δ: 105.6 (C_4′_), 119.0 (C≡N), 120.6 (C_3′_, C_5′_), 122.2 (C_3_), 123.7 (C_1_), 124.2 (C_5_), 128.3 (C_6_), 128.9 (C_4′′_), 129.1 (C_5′′_), 132.2 (C_4_), 132.3 (C_3′′_), 133.1 (C_2′_, C_6′_), 137.7 (C_2′′_), 139.4 (C_2_), 143.1 (C_1′_), 159.5 (CONH), 167.6 (NHCO); DIP-MS (EI) *m*/*z*: 347 [M]^+^.

*N*-(4′-Cyanomethylphenyl)-2-((thiophen-2′′-yl)carbonylamino)benzamide (**23c**). Yield: 88%; m.p.: 221–222 °C; ^1^H NMR (400 MHz, DMSO-*d*_6_) δ: 4.02 (2H, s, CH_2_), 7.25 (1H, dd, *J* = 5.0, 4.0 Hz, H_4′′_), 7.29 (1H, dt, *J* = 7.7, 1.1 Hz, H_5_), 7.36 (2H, d, *J* = 8.0 Hz, H_3′_ + H_5′_), 7.61 (1H, dt, *J* = 8.0, 1.2 Hz, H_4_), 7.74 (2H, d, *J* = 8.0 Hz, H_2′_ + H_6′_), 7.75 (1H, d, *J* = 7.6 Hz, H_6_), 7.89 (1H, d, *J* = 5.0 Hz, H_5′′_), 7.93 (1H, dd, *J* = 7.8, 1.2 Hz, H_3_), 8.35 (1H, dd, *J* = 8.2, 1.2 Hz, H_3′′_), 10.60 (1H, s, NHCO), 11.66 (1H, s, CONH); ^13^C NMR (100 MHz, DMSO-*d*_6_) δ: 21.9 (CH_2_), 119.3 (C≡N), 121.4 (C_3_), 121.5 (C_2′_, C_6′_), 122.8 (C_1_), 123.4 (C_5_), 126.9 (C_4′_), 128.4 (C_3′_, C_5′_), 128.8 (C_6_), 129.0 (C_4′′_), 129.0 (C_5′′_), 132.2 (C_4_), 132.3 (C_3′′_), 137.9 (C_2′′_), 138.3 (C_1_′), 139.5 (C_2_), 159.4 (CONH), 167.3 (NHCO); DIP-MS (EI) *m*/*z*: 361 [M]^+^.

#### 3.1.11. General Synthetic Procedures for **21d**–**23d**

The nitriles **21c**–**23c** (0.86 mmol) were added in absolute ethanol (25 mL) and 1,4-dioxane (5 mL) mixture and added with triethylamine (2.59 mmol) and then refluxed to dissolve. The reaction mixture was added with hydroxylamine·HCl (3.45 mmol) and refluxed for 4–6 h. The ethanol and trimethylamine were removed under reduced pressure and the residue was poured with water to form the precipitate. The obtained precipitate was filtrated, washed with cold water to afford pure white or pale yellow compounds **21d**–**23d**.

*N*-(3′-Amidoximephenyl)-2-((thiophen-2′′-yl)carbonylamino)benzamide (**21d**). Yield: 94%; m.p.: 230–232 °C; ^1^H NMR (400 MHz, DMSO-*d*_6_) δ: 5.78 (2H, s, NH_2_), 7.25 (1H, dd, *J* = 5.0, 4.0 Hz, H_4′′_), 7.29 (1H, dt, *J* = 7.7, 1.1 Hz, H_5_), 7.38 (1H, t, *J* = 8.0 Hz, H_5′_), 7.43 (1H, dt, *J* = 7.7, 1.4 Hz, H_4′_), 7.61 (1H, dt, *J* = 7.6, 2.4 Hz, H_4_), 7.68–7.71 (2H, m, H_6_ + H_6′_), 7.89 (1H, dd, *J* = 5.0, 1.1 Hz, H_5′′_), 7.95 (1H, dd, *J* = 7.6, 1.2 Hz, H_3_), 8.04 (1H, t, *J* = 1.7 Hz, H_2′_), 8.38 (1H, dd, *J* = 4.0, 0.8 Hz, H_3′′_), 9.65 (1H, s, =NOH), 10.59 (1H, s, NHCO), 11.71 (1H, s, CONH); ^13^C NMR (100 MHz, DMSO-*d*_6_) δ: 116.6 (C_2′_), 122.8 (C_3_), 123.4 (C_1_), 124.6 (C_5_), 124.9 (C_3′_), 129.2 (C_4′′_), 129.3 (C_5′′_), 129.8 (C_6_), 132.1 (C_4_), 132.8 (C_3′′_), 134.8 (C_5′_), 138.1 (C_2′′_), 138.7 (C_1′_), 139.3 (C_2_), 159.1 (CONH), 167.7 (NHCO); FAB-MS *m*/*z*: 363 [M + 1 − H_2_O]^+^, 380 [M]^+^, 381 [M + 1]^+^, 403 [M + Na]^+^.

*N*-(4′-Amidoximephenyl)-2-(thiophen-2′′-ylcarbonylamino)benzamide (**22d**). Yield: 52%; m.p.: 241–243 °C; ^1^H NMR (400 MHz, DMSO-*d*_6_) δ: 5.40 (2H, s, NH_2_), 7.20–7.29 (4H, m, H_5_ + H_2′_ + H_6′_ + H_4′′_), 7.56–7.64 (3H, m, H_4_ + H_3′_ + H_5′_), 7.80 (1H, d, *J* = 7.6 Hz, H_6_), 7.88 (1H, d, *J* = 4.0 Hz, H_5′′_), 7.96 (1H, d, *J* = 7.6 Hz, H_3_), 8.30 (1H, d, *J* = 8.4 Hz, H_3′′_), 8.80 (1H, s, =NOH), 10.20 (1H, s, NHCO), 11.12 (1H, s, CONH); ^13^C NMR (100 MHz, DMSO-*d*_6_) δ: 120.6 (C_2′_, C_6′_), 122.5 (C_3_), 123.2 (C_1_), 124.3 (C_5_), 128.8 (C_3′_, C_5′_), 128.7 (C_6_), 129.0 (C_4′′_, C_5′′_), 133.1 (C_4_), 134.2 (C_3′′_), 136.6 (C_1′_), 138.0 (C_2′′_), 138.7 (C_2_), 150.2 (C=NOH), 154.3 (CONH), 165.3 (NHCO); FAB-MS *m*/*z*: 363 [M + 1 − H_2_O]^+^, 380 [M]^+^, 381 [M + 1]^+^.

*N*-(4′-Amidoximemethylphenyl)-2-(thiophen-2′′-ylcarbonylamino)benzamide (**23d**). Yield: 89%; m.p.: 209–211 °C; ^1^H NMR (400 MHz, DMSO-*d*_6_) δ: 3.26 (2H, s, CH_2_), 5.43 (2H, s, NH_2_), 7.19–7.27 (4H, m, H_5_ + H_3′_ + H_5′_ + H_4′′_), 7.56–7.62 (3H, m, H_4_ + H_2_ + H_6′_), 7.73 (1H, d, *J* = 2.8 Hz, H_6_), 7.89 (1H, d, *J* = 4.0 Hz, H_5′′_), 7.93 (1H, d, *J* = 7.6 Hz, H_3_), 8.39 (1H, d, *J* = 8.4 Hz, H_3′′_), 8.92 (1H, s, =NOH), 10.50 (1H, s, NHCO), 11.79 (1H, s, CONH); ^13^C NMR (100 MHz, DMSO-*d*_6_) δ: 36.7 (CH_2_), 121.1 (C_2′_, C_6′_), 122.5 (C_3_), 123.2 (C_1_), 128.4 (C_4′_), 128.8 (C_3′_, C_5_′), 128.9 (C_6_), 128.9 (C_4′′_, C_5′′_) 132.3 (C_4_), 133.9 (C_3′′_), 136.6 (C_1′_), 138.4 (C_2′′_), 139.7 (C_2_), 152.2 (C=NOH), 159.3 (CONH), 167.3 (NHCO); FAB-MS *m*/*z*: 377 [M + 1 − H_2_O]^+^, 394 [M]^+^, 395 [M + 1]^+^, 417 [M + Na]^+^.

#### 3.1.12. General Synthetic Procedures for **21**–**23**

Amidoximes **21d**–**23d** (0.80 mmol) were dissolved in anhydrous dichloromethane (15 mL) and added with triethylamine (2.37 mmol) at room temperature and acetyl chloride (0.9 mmol) was added at 0 °C. The mixture was reacted for 1–2 h at room temperature and ice water was added. The aqueous layer was extracted with dichloromethane (3 × 40 mL) and the organic phase was washed with water, saturated sodium bicarbonate and water. The organic layer was dried with anhydrous MgSO_4_, filtrated, and concentrated in reduced pressure to give the acetyl amidoximes (**21e**–**23e**), which were used in next reaction without purification. The acetyl amidoximes (1.0 eq) was added to a suspension of 10% Pd-C in absolute ethanol (10 mL) and c-HCl (1.0 eq), and carried out catalytic hydrogenatin reaction for 2 h at 60 psi, 45 °C. The reaction mixture was filtrated on celite pad and the filtrate was evaporated under reduced pressure to give a crude oily residue, which was purified to yield white compounds **21**–**23** by medium pressure liquid chromatography.

*N*-(3′-Amidinophenyl)-2-((thiophen-2′′-yl)carbonylamino)benzamide·HCl (**21**). Yield: 32%; m.p.: 262–264 °C; ^1^H NMR (400 MHz, DMSO-*d*_6_) δ: 7.24 (1H, dd, *J* = 5.0, 4.0 Hz, H_4′′_), 7.33 (1H, t, *J* = 8.0 Hz, H_5_), 7.53 (1H, d, *J* = 8.0 Hz, H_4′_), 7.60–7.65 (2H, m, H_4_ + H_5′_), 7.79 (1H, dd, *J* = 3.7, 0.8 Hz, H_6_), 7.89 (1H, dd, *J* = 5.0, 0.8 Hz, H_5′′_), 7.92 (1H, d, *J* = 8.0 Hz, H_6′_), 8.00 (1H, d, *J* = 8.3 Hz, H_3_), 8.21 (1H, s, H_2′_), 8.26 (1H, dd, *J* = 8.0, 0.8 Hz, H_3′′_), 9.08 (2H, br s, hydrogens of amidinium chloride), 9.36 (2H, br s, hydrogens of amidine·HCl), 10.86 (1H, s, NHCO), 11.45 (1H, s, CONH); ^13^C NMR (100 MHz, DMSO-*d*_6_) δ: 120.0 (C_2′_), 122.0 (C_3_), 123.5 (C_1_), 123.7 (C_5_), 125.7 (C_6′_), 128.3 (C_6_), 128.9 (C_4′′_), 129.0 (C_5′′_), 129.5 (C_4′_), 132.3 (C_4_), 132.4 (C_3′′_), 137.9 (C_2′′_), 139.2 (C_1′_), 139.5 (C_2_), 159.4 (carbon of amidine), 166.0 (CONH), 167.6 (NHCO); FAB-MS *m*/*z*: 388 [M − Cl + Na]^+^, 365 [M − Cl]^+^; HRMS (FAB): calcd for C_19_H_17_N_4_O_2_S [M + H]^+^ 365.1072, found 365.1084.

*N*-(4′-Amidinophenyl)-2-((thiophen-2′′-yl)carbonylamino)benzamide·HCl (**22**). Yield: 51%; m.p.: 211–213 °C; ^1^H NMR (400 MHz, DMSO-*d*_6_) δ: 7.24 (1H, dd, *J* = 5.0, 4.0 Hz, H_4′′_), 7.30 (1H, t, *J* = 8.0 Hz, H_5_), 7.61 (1H, dt, *J* = 8.0, 1.2 Hz, H_4_), 7.82 (1H, dd, *J* = 4.0, 1.2 Hz, H_6_), 7.88 (2H, d, *J* = 8.0 Hz, H_3′_ + H_5′_), 7.88 (1H, d, *J* = 4.0, 1.2 Hz, H_5′′_), 7.94 (1H, dd, *J* = 8.0, 1.2 Hz, H_3_), 7.97 (2H, d, *J* = 8.0 Hz, H_2′_ + H_6′_), 8.18 (1H, dd, *J* = 8.0, 0.8 Hz, H_3′′_), 9.36 (4H, br s, hydrogens of amidinium chloride), 10.77 (1H, s, NHCO), 11.53 (1H, s, CONH); ^13^C NMR (100 MHz, DMSO-*d*_6_) δ: 120.2 (C_2′_, C_6′_), 122.4 (C_3_), 122.5 (C_4′_), 123.6 (C_1_), 124.4 (C_5_), 128.3 (C_6_), 128.9 (C_3′_, C_5′_, C_4′′_), 129.2 (C_5′′_), 132.1 (C_4_), 132.2 (C_3′′_), 137.9 (C_2′′_), 139.7 (C_2_), 143.9 (C_1′_), 159.6 (carbon of amidine), 164.9 (CONH), 167.5 (NHCO); FAB-MS *m*/*z*: 365 [M − Cl]^+^; HRMS (FAB): calcd for C_19_H_17_N_4_O_2_S [M + H]^+^ 365.1072, found 365.1089.

*N*-(4′-Amidinomethylphenyl)-2-((thiophen-2′′-yl)carbonylamino)benzamide·HCl (**23**). Yield: 42%; m.p.: 172–174 °C; ^1^H NMR (400 MHz, DMSO-*d*_6_) δ: 3.69 (2H, s, CH_2_), 7.24 (1H, dd, *J* = 5.0, 4.0 Hz, H_4′′_), 7.30 (1H, dt, *J* = 7.7, 1.1 Hz, H_5_), 7.42 (2H, d, *J* = 8.0 Hz, H_3′_ + H_5′_), 7.61 (1H, dt, *J* = 8.0, 1.2 Hz, H_4_), 7.72 (2H, d, *J* = 8.0 Hz, H_2′_+ H_6′_), 7.75 (1H, dd, *J* = 4.0, 1.2 Hz, H_6_), 7.89 (1H, dd, *J* = 4.0, 1.2 Hz, H_5′′_), 7.92 (1H, dd, *J* = 8.0, 1.2 Hz, H_6_), 8.33 (1H, d, *J* = 8.0 Hz, H_3′′_), 8.92 (4H, br s, hydrogens of amidinium chloride), 10.61 (1H, s, NHCO), 11.64 (1H, s, CONH); ^13^C NMR (100 MHz, DMSO-*d*_6_) δ: 121.4 (C_2′_, C_6′_), 121.5 (C_3_), 123.0 (C_1_), 123.4 (C_5_), 128.4 (C_6_), 128.8 (C_4′′_), 129.0 (C_5′′_), 129.1 (C_3′_, C_5′_), 130.7 (C_4′_), 132.2 (C_4_), 132.3 (C_3′′_), 138.0 (C_2′′_), 138.2 (C_1′_), 139.6 (C_2_), 159.4 (carbon of amidine), 167.3 (CONH), 169.1 (NHCO); FAB-MS *m*/*z*: 379 [M − Cl]^+^; HRMS (FAB): calcd for C_20_H_19_N_4_O_2_S [M + H]^+^ 379.1229, found 379.1243.

#### 3.1.13. General Synthetic Procedures for **24b**–**26b**

To a solution of **11a**–**13a** (1.54 mmol) in ethanol (40 mL) was added with hydroxylamine hydrochloride (300 mg, 4.60 mmol) and triethylamine (0.5 mL) at room temperature. The reaction mixture was refluxed for 12 h and the following procedures were same as synthetic procedure for **21b**–**23b**. Compounds **24b**–**26b** were recrystallized with dichloromethane and *n*-hexane mixture to afford as a white powder, respectively.

*N*-(3′-Amidoximino)-2-(4"-methylphenylsulfonamido)benzamide (**24b**). Yield: 85%; m.p: 182–184 °C; ^1^H NMR (400 MHz, CDCl_3_) δ: 2.23 (3H, s, CH_3_), 5.05 (2H, s, NH_2_), 6.61 (1H, s, =NOH), 7.02–7.12 (4H, m, H_3_ + H_4_ + H_5_ + H_5′_), 7.37 (2H, d, *J* = 8.0 Hz, H_3′′_ + H_5′′_), 7.50–7.60 (3H, m, H_6_ + H_4′_ + H_6′_), 7.61 (2H, d, *J* = 8.0 Hz, H_2′′_ + H_6′′_), 7.85 (1H, s, H_2′_), 7.91 (1H, s, NHSO_2_), 8.26 (1H, s, CONH); ^13^C NMR (100 MHz, CDCl_3_) δ: 21.4 (CH_3_), 118.3 (C_2′_), 121.9 (C_6′_), 122.2 (C_3_), 122.5 (C_1_), 124.0 (C_5_), 124.1 (C_3′_), 127.2 (C_2′′_, C_6′′_), 129.4 (C_3′′_, C_5′′_), 129.7 (C_6_), 132.9 (C_4_), 133.1 (C_5′_), 136.2 (C_1′′_), 137.5 (C_4′′_), 138.6 (C_1′_), 143.9 (C_2_), 158.8 (C=NOH), 166.8 (C=O); GC-MS (EI) *m*/*z*: 409 [M + 1-NH_2_]^+^, 392 [M + 1 − NH_2_OH]^+^.

*N*-(4′-Amidoximino)-2-(4"-methylphenylsulfonamido)benzamide (**25b**). Yield: 74%; m.p: 213–215 °C; ^1^H NMR (400 MHz, (CD_3_)_2_CO) δ: 2.26 (3H, s, CH_3_), 6.60 (1H, s, =NOH), 7.19 (3H, m, H_4_ + H_3′′_ + H_5′′_), 7.54 (1H, t, *J* = 7.2 Hz, H_5_), 7.62 (2H, d, *J* = 7.6 Hz, H_2′′_ + H_6′′_), 7.69 (1H, d, *J* = 8.4 Hz, H_3_), 7.75–7.88 (3H, m, H_6_ + H_2′_ + H_6′_), 7.98 (2H, d, *J* = 8.4 Hz, H_3′_ + H_5′_), 9.72 (1H, s, NHSO_2_), 10.50 (1H, s, CONH); ^13^C NMR (100 MHz, (CD_3_)_2_CO) δ: 21.4 (CH_3_), 120.9 (C_3_), 122.9 (C1), 124.2 (C_5_), 125.0 (C_4′_), 128.0 (C_2′_, C_6′_), 129.2 (C_3′_, C_5′_, C_2′′_, C_6′′_), 130.6 (C_3′′_, C_5′′_), 131.2 (C_6_), 133.6 (C_4_), 137.3 (C_1′′_), 139.5 (C_4′′_), 142.0 (C_1′_), 144.8 (C_2_), 168.2 (C=NOH), 168.3 (C=O); GC-MS (EI) *m*/*z*: 409 [M + 1-NH_2_]^+^, 392 [M + 1 − NH_2_OH]^+^.

*N*-(4′-Amidoximinobenzyl)-2-(4"-methylphenylsulfonamido)benzamide (**26b**). Yield: 63%; m.p: 70–74 °C; ^1^H NMR (400 MHz, DMSO-*d*_6_) δ: 2.29 (3H, s, CH_3_), 3.52 (2H, s, CH_2_), 7.22 (1H, t, *J* = 7.2 Hz, H_4_), 7.25 (2H, d, *J* = 7.6 Hz, H_3′_ + H_5′_), 7.26 (2H, d, *J* = 8.4 Hz, H_3′′_ + H_5′′_), 7.42–7.50 (2H, m, H_3_ + H_5_), 7.52 (2H, d, *J* = 7.6 Hz, H_2′_ + H_6′_), 7.61 (2H, d, *J* = 8.4 Hz, H_2′′_ + H_6′′_), 7.78 (1H, d, *J* = 8.0 Hz, H_6_), 8.49 (2H, br s, NH_2_), 9.50 (1H, br s, =NOH), 10.35 (1H, s, NHSO_2_), 10.75 (1H, s, CONH); ^13^C NMR (100 MHz, DMSO-*d*_6_) δ: 21.4 (CH_3_), 120.9 (C_3_), 122.9 (C_1_), 124.2 (C_5_), 125.0 (C_4′_), 128.0 (C_2′_, C_6′_), 129.2 (C_3′_, C_5′_, C_2′′_, C_6′′_), 130.6 (C_3′′_, C_5′′_), 131.2 (C_6_), 133.6 (C_4_), 137.3 (C_1′′_), 139.5 (C_4′′_), 142.0 (C_1′_), 144.8 (C_2_), 168.2 (C=NOH), 168.3 (C=O); GC-MS (EI) *m*/*z*: 423 [M + 1 − NH_2_]^+^, 406 [M + 1 − NH_2_OH]^+^.

#### 3.1.14. General Experimental Procedures for **24**–**26**

To a solution of amidoximes **24b**–**26b** (1.18 mmol) in anhydrous dichloromethane (10 mL) was added triethylamine (1.18 mmol) and acetyl chloride (1.20 mmol) at 0 °C. The following procedures were same as synthetic procedures for acetylated amidoximes **21c**–**23c**. To a suspention of 10% Pd-C in absolute ethanol (50 mL) was added **21c**–**23c** (1.12 mmol) and c-HCl (1.12 mmol) and the reaction mixture was shaken on Paar hydrogenation apparatus for 12 h at 50 psi, 45 °C and filtrated on celite pad. The filtrate was evaporated under reduced pressure to provide a crude compound, which was purified to give white compounds **24**–**26** by column chromatography.

*N*-(3′-Amidinophenyl)-2-(4"-methylphenylsulfonamido)benzamide·HCl (**24**). Yield: 37% m.p: 216–218 °C; ^1^H NMR (400 MHz, DMSO-*d*_6_) δ: 2.29 (3H, s, CH_3_), 7.20–7.29 (3H, m, H_4_ + H_3′′_ + H_5′′_), 7.39 (1H, d, *J* = 6.8 Hz, H_3_), 7.55 (1H, t, *J* = 8.0 Hz, H_5_), 7.58–7.68 (4H, m, H_3′_ + H_5′_ + H_2′′_ + H_6′′_), 7.86 (1H, d, *J* = 7.6 Hz, H_6_), 7.92 (1H, d, *J* = 6.8 Hz, H_6′_), 8.24 (1H, s, H_2′_), 9.28 (2H, br s, hydrogens of amidinium chloride), 9.46 (2H, s, hydrogens of amidinium chloride), 10.57 (1H, s, NHSO_2_), 10.80 (1H, s, CONH); ^13^C NMR (100 MHz, CD_3_OD) δ: 20.2 (CH_3_), 119.4 (C_2′_), 121.3 (C_3_), 121.8 (C_1_), 122.4 (C5), 123.0 (C_6′_), 124.2 (C_4′_), 125.8 (C_3′_), 126.8 (C_2′′_, C_6′′_), 126.9 (C_3′′_, C_5′′_), 129.1 (C_4_), 129.2 (C_6_), 129.8 (C_5′_), 130.6 (C_1′_), 132.3 (C_1′′_), 139.9 (C_4′′_), 142.9 (C_2_), 167.3 (C=NH), 167.6 (C=O); DIP-MS (EI) *m*/*z*: 392 [M – HCl − NH_2_]^+^; HRMS (FAB): calcd for C_21_H_21_N_4_O_3_S [M + H]^+^ 409.1334, found 409.1347.

*N*-(4′-Amidinophenyl)-2-(4"-methylphenylsulfonamido)benzamide·HCl (**25**). Yield: 43%; m.p: 250–252 °C; ^1^H NMR (400 MHz, DMSO-*d*_6_) δ: 2.27 (3H, s, CH_3_), 7.23–7.32 (3H, m, H_4_ + H_3′′_ + H_5′′_), 7.35 (1H, d, *J* = 8.0 Hz, H_3_), 7.50 (1H, t, *J* = 8.4 Hz, H_5_), 7.60 (2H, d, *J* = 8.0 Hz, H_2′′_ + H_6′′_), 7.78 (1H, dd, *J* = 8.0, 1.2 Hz, H_6_), 9.10 (2H, br s, hydrogens of amidinium chloride), 9.35 (2H, br s, hydrogens of amidinium chloride), 10.34 (1H, s, NHSO_2_), 10.73 (1H, s, CONH); ^13^C NMR (100 MHz, DMSO-*d*_6_) δ: 20.9 (CH_3_), 120.1 (C_2′_, C_6′_), 122.2 (C_3_), 122.6 (C_1_), 124.5 (C_5_), 125.4 (C_4′_), 126.8 (C_3′_, C_5′_), 128.3 (C_2′′_, C_6′′_), 129.3 (C_6_), 129.5 (C_3′′_, C_5′′_), 132.4 (C_4_), 136.0 (C_1′′_), 136.7 (C_4′′_), 143.5 (C_1′_), 143.6 (C_2_), 164.8 (C=NH), 166.9 (C=O); DIP-MS (EI) *m*/*z*: 392 [M – HCl − NH_2_]^+^; HRMS (FAB): calcd for C_21_H_21_N_4_O_3_S [M + H]^+^ 409.1334, found 409.1348.

*N*-(4′-Amidinomethylphenyl)-2-(4"-methylphenylsulfonamido)benzamide·HCl (**26**). Yield: 33%; m.p.: 172–173 °C; ^1^H NMR (400 MHz, DMSO-*d*_6_) δ: 2.28 (3H, s, CH_3_), 3.67 (2H, s, CH_2_), 7.23 (1H, t, *J* = 8.0 Hz, H_4_), 7.26 (2H, d, *J* = 8.0 Hz, H_3′′_ + H_5′′_), 7.41–7.53 (4H, m, H_3_ + H_5_ + H_3′,_+ H_5′_), 7.60 (2H, d, *J* = 8.4 Hz, H_2′_ + H_6′_), 7.66 (2H, d, *J* = 8.8 Hz, H_2′′_ + H_6′′_), 7.81 (1H, dd, *J* = 8.0, 1,2 Hz, H_6_), 8.79 (2H, br s, hydrogens of amidinium chloride), 9.28 (2H, br s, hydrogens of amidinium chloride), 10.44 (1H, s, NHCO), 10.65 (1H, s, CONH); ^13^C NMR (100 MHz, DMSO-*d*_6_) δ: 20.9 (CH_3_), 37.1 (CH_2_), 121.2 (C_2′_, C_6′_), 123.8 (C_3_), 124.1 (C_1_), 126.8 (C_3′_, C_5′_), 128.3 (C_6_), 129.2 (C_2′′_, C_6′′_), 129.8 (C_3′′_, C_5′′_), 129.9 (C_4_), 132.4 (C_4′_), 135.9 (C_1′_), 137.3 (C_1′′_), 137.7 (C_4′′_), 143.7 (C_2_), 166.7 (C=NH), 169.2 (NHCO); DIP-MS (EI) *m*/*z*: 406 [M – HCl − NH_2_]^+^; HRMS (FAB): calcd for C_22_H_23_N_4_O_3_S [M + H]^+^ 423.1491, found 423.1478.

### 3.2. Biology

#### 3.2.1. Reagents and Instruments

Heparin was purchased from Sigma (St. Louis, MO, USA) and TNF-α was purchased from Abnova (Taipei, Taiwan). The molecular weight of heparin used as positive control in our experiments is 3500. Anti-tissue factor (TF) antibody, rivaroxaban, and argatroban were purchased from Santa Cruz Biotechnology, Inc. (Dallas, TX, USA). Thromboxane A_2_ (TXA_2_) analog and U46619 were purchased from Calbiochem-Novabiochem Corp. (San Diego, CA, USA). Prothrombin, thrombin, Factor VIIa, X, and Xa were purchased from Haematologic Technologies (Essex Junction, VT, USA) and APTT-XL and thromboplastin-D were purchased from Fisher Diagnostics (Middletown, VA, USA). S-2222 and S-2238 were purchased as substrate of FXa and thrombin from ChromogenixAB (Mölndal, Sweden). Aggregometer (Chronlog, Havertown, PA, USA), thrombotimer (Behnk Elektronik, Norderstedt, Germany), microplate reader (Tecan Austria GmbH, Grödig, Austria), and spectrophotometer (TECAN, Männedorf, Switzerland) were used.

#### 3.2.2. Preparation of Plasma

Blood samples were collected in the morning from 10 healthy volunteers after fasting overnight (aged between 24 and 28 years, four males and six females) without cardiovascular disorders, allergy and lipid or carbohydrate metabolism disorders, untreated with drugs. All volunteers gave written informed consent before participation. Human blood was collected into sodium citrate (0.32% final concentration) and immediately centrifuged (2000× *g*, 15 min) to prepare plasma. The study protocol was approved by the Institutional Review Board of Kyungpook National University Hospitals (Daegu, Republic of Korea).

#### 3.2.3. In Vitro Anticoagulant Assay

According to the manufacturer’s instructions, as described previously [41], aPTT and PT of compounds **5**, **9**, and **21**–**23** were measured by a Thrombotimer (Behnk Elektronik, Norderstedt, Germany). The aPTT and PT results are expressed in seconds and PT results are also expressed as INR. INR (International Normalized Rations) = (PT sample/PT control) ^ISI^, ISI = international sensitivity index.

#### 3.2.4. In Vivo Bleeding Time

According to procedure reported by Dejana et al. [42], tail bleeding times were measured. Briefly, ICR mice were fasted overnight before experiments. One hour after intravenous administration of **5**, **9**, and **21**–**23**, tails of mice were transected at 2 mm from their tips. Bleeding time was defined as the time elapsed until bleeding stopped. When the bleeding time exceeded 10 min, in vivo bleeding time was recorded as 10 min. All animals were treated in accordance with the guidelines for the Care and Use of Laboratory Animals issued by Kyungpook National University.

#### 3.2.5. Thrombin Activity Assay

According to procedure previously described [22], compounds **5**, **9**, and **21**–**23** were dissolved in 50 nM Tris-HCl buffer (pH 7.4) containing 7.5 mM ethylenediaminetetraacetic acid (EDTA) and added with 150 mM NaCl. After incubation at 37 °C for 2 min, thrombin solution (150 µL; 10 U/ mL) was added and incubated at 37 °C for 1 min. S-2238 as substrate (150 µL; 1.5 mM) solution was added and then absorbance was measured at 405 nm for 2 min using a spectrophotometer (TECAN, Männedorf, Switzerland).

#### 3.2.6. Factor Xa Activity Assay 

Factor Xa activity assay was carried out in the same method as the thrombin activity assay, but using FXa (150 µL; 1 U/mL) and S-2222 as FXa substrate.

#### 3.2.7. Cell Culture

Primary HUVECs were purchased from Cambrex Bio Science (Charles City, IA, USA) and were maintained by a previously described method [43,44]. Briefly, cells were cultured until confluent at 37 °C in 5% CO_2_ in EBM-2 basal media supplemented from Cambrex Bio Science (Charles City, IA, USA).

#### 3.2.8. Thrombin Generation on the Surfaces of Human Umbilical Vein Endothelial Cells (HUVECs)

The thrombin generation of HUVECs was quantitated as previously described [40]. Briefly, HUVECs were pre-incubated with synthesized compounds (300 µL) dissolved in 50 mM Tris-HCl buffer, 100 pM FVa, and 1 nM FXa for 10 min, and then added with prothrombin to a final concentration of 1 µM. After 10 min, Each 10 µL of triplicate samples was transferred to a 96-well plate containing 40 µL of 0.5 M EDTA in Tris-buffered saline per well. Activated prothrombin was determined by measuring the rate of hydrolysis of S2238 at 405 nm. Standard curves were prepared using amounts of purified thrombin.

#### 3.2.9. Factor Xa Generation on the Surfaces of HUVECs

The FXa generation of HUVECs was measured as previously described [41]. The amounts of FXa generated by indicated concentrations of **5**, **9**, and **21**–**23** were measured at 405 nm over 2 min by a microplate reader. Initial rates of color development were converted into FXa concentrations using a standard curve prepared with known dilutions of purified human FXa.

#### 3.2.10. Thrombin-Catalyzed Fibrin Polymerization

Thrombin-catalyzed polymerization was monitored turbidity at 360 nm every 6 s for 20 min by using a spectrophotometer (TECAN, Männedorf, Switzerland) at ambient temperature. Both control plasma and plasma incubated with compounds **5**, **9**, and **21**–**23** were diluted three times in TBS 50 mM Tris-buffered saline and clotted with thrombin (final concentration-0.5 U/mL). The maximum rate of fibrin polymerization (Vmax, ΔmOD/min) was recorded for each absorbance curve. All experiments were performed in triplicate.

#### 3.2.11. Platelet Aggregation

The platelet aggregation assay was measured following to the previously reported method [44]. Washed plasma was pre-incubated with the indicated concentration of compounds **5**, **9**, and **21**–**23** for 3 min, and then stimulated with thrombin (0.1 U/mL, Sigma) and U46619 (2 µM) in 0.9% saline for 5 min. Platelet aggregations were performed in an aggregometer (Chronolog, Havertown, PA, USA).

#### 3.2.12. Cell Viability Assay

The colorimetric MTT was used for counting the number of live cells to determine cell viability. Cells (5 × 10^3^/well) were grown in a 96-well plate at 37 °C and after incubation for 24 h, cells were washed with fresh medium, and treated with compounds **5**, **9**, and **21**–**23**. After a 48 h incubation period, cells were washed and 100 µL of 1 mg/mL MTT was added, followed by incubation for 4 h. Finally, the resultant formazan salt was dissolved in 150 µL DMSO and the absorbance intensity determinded by a microplate reader (Tecan Austria GmbH, Grödig, Austria) with a reference wavelength of 540 nm using. Data were expressed as means ± SEM at least three independent experiments.

#### 3.2.13. Statistical Analysis

Data are expressed as means ± SEM of at least three independent experiments. Statistical significance (*p* < 0.05) was determiced using the Student’s *t*-test.

## 4. Conclusions

Non-amidino *N*^2^-thiophenecarbonyl- and *N*^2^-tosylanthranilamides **1**–**20**, and amidino *N*^2^- thiophenecarbonyl- and *N*^2^-tosylanthranilamides **21**–**26** synthesized and evaluated against PT and aPTT in vitro*.* Compounds **5**, **9**, and **21**–**23** showed prolongation in aPTT in vitro and ex vivo, and in vivo bleeding time. Compounds 5 and 21–23 exhibited prolongation in PT in vitro and ex vivo. The activities of FXa and thrombin as well as the generation of thrombin and FXa in HUVECs were dose-dependently inhibited but weak. These compounds inhibited thrombin-catalyzed fibrin polymerization and platelet aggregation induced by U46619. Any sulfonamide group containing compounds **11**–**20**, **24**, and **26** did not exhibit prolongation of PT and aPTT, but 4-amidino derivative **25** showed high aPTT. The anticoagulant activity was governed to a great extent by an amidine group at the meta or para position of phenyl ring and thiophenecarbonyl linker of **21**–**23**.

## Figures and Tables

**Table 1:** Anticoagulant activities of non-amidine compounds **1**–**20** at 30 µg/mL.

Compound No.	aPPT (s)	PT (s)	Compound No.	aPTT (s)	PT (s)
DMSO ^1^	39.0	11.1	11	40.2	11.8
1	44.5	14.1	12	41.2	11.7
2	37.4	13.2	13	42.3	10.9
3	41.6	11.8	14	38.6	13.6
4	41.7	11.6	15	35.8	11.3
5	60.6	12.8	16	36.2	11.0
6	39.9	12.2	17	41.5	11.7
7	44.9	12.2	18	38.4	11.0
8	59.3	12.7	19	35.8	10.7
9	65.1	12.5	20	33.6	10.8
10	53.3	14.7	Heparin ^2^	85 (30 µM)	34 (30 µM)

^1^ Dimethyl sulfoxide (DMSO) was used as the negative control. ^2^ The molecular weight of heparin used as positive control is 3500. aPTT: activated partial thromboplastin time, PT: prothrombin time.

**Table 2:** In vitro anticoagulant activities of **5** and **9**.

Compound No.	Dose	aPTT (s)	PT (s)	PT (INR)
Control	saline	23.6 ± 0.6	12.4 ± 0.4	1.00
**5**	5 µM	23.7 ± 0.2	12.2 ± 0.4	0.96
	10 µM	24.2 ± 0.8	12.6 ± 0.6	1.04
	20 µM	33.2 ± 0.5 *	14.0 ± 0.3 *	1.34 *
	30 µM	40.4 ± 0.6 *	15.2 ± 0.5 *	1.63 *
	40 µM	58.5 ± 0.5 *	18.5 ± 0.4 *	2.61 *
	50 µM	60.5 ± 0.7 *	19.1 ± 0.6	2.78 *
**9**	5 µM	23.2 ± 0.4	12.0 ± 0.2	0.92
	10 µM	23.4 ± 0.5	12.2 ± 0.5	0.96
	20 µM	37.6 ± 0.3 *	12.0 ± 0.2	0.92
	30 µM	44.1 ± 0.4 *	11.6 ± 0.6	0.85
	40 µM	65.6 ± 0.6 *	12.7 ± 0.5	1.06
	50 µM	63.2 ± 0.8 *	13.0 ± 0.8	1.12
Heparin ^1^	20 µM	53.3 ± 0.5 *	21.6 ± 0.4 *	3.79 *
	30 µM	62.5 ± 0.8 *	27.2 ± 0.6 *	6.59 *

^1^ The molecular weight of heparin used as positive control in our experiments is 3500. Each value represents the means ± standard error of mean (SEM) (*n* = 5). * *p* < 0.05.

**Table 3:** In vivo Bleeding time of **5** and **9**.

Compound No.	Dose	Tail Bleeding Time (s)
Control	saline	30.8 ± 0.8
**5**	24.4 µg/mouse	37.4 ± 1.0 *
	32.6 µg/mouse	53.7 ± 1.2 *
	40.8 µg/mouse	61.8 ± 1.2 *
**9**	23.2 µg/mouse	48.2 ± 1.6 *
	30.9 µg/mouse	62.2 ± 1.4 *
	38.7 µg/mouse	65.4 ± 1.6 *
Heparin ^1^	140.0 µg/mouse 210.0 µg/mouse	51.2 ± 1.2 *
		68.4 ± 1.2 *

^1^ The molecular weight of heparin used as positive control in our experiments is 3500. Each value represents the means ± SEM (*n* = 5). * *p* < 0.05.

**Table 4:** Ex vivo clotting time of **5** and **9**.

Compound No.	Dose	aPTT (s)	PT (s)	PT (INR)
Control	saline	32.2 ± 0.8	12.8 ± 0.4	1.00
**5**	24.4 µg /mouse	38.4 ± 0.8 *	14.8 ± 0.4 *	1.42 *
	32.6 µg/mouse	51.2 ± 1.4 *	17.4 ± 0.8 *	2.09 *
	40.8 µg/mouse	60.8 ± 1.2 *	17.2 ± 0.7 *	2.03 *
**9**	23.2 µg/mouse	42.2 ± 0.6 *	12.6 ± 0.8	0.96
	30.9 µg/mouse	57.4 ± 1.2 *	13.0 ± 0.6	1.04
	38.7 µg/mouse	61.2 ± 1.0 *	12.4 ± 0.8	0.93
Heparin ^1^	140.0 µg/mouse	63.4 ± 0.9 *	25.4 ± 0.4 *	5.18 *
	210.0 µg/mouse	75.4 ± 1.2 *	31.4 ± 0.6 *	8.62 *

^1^ The molecular weight of heparin used as positive control in our experiments is 3500. Each value represents the means ± SEM (*n* = 5). * *p* < 0.05.

**Table 5:** Anticoagulant activities of amidine compounds **21**–**26** at 30 µg/mL.

Compound No.	aPTT (s)	PT (s)	Compound No.	aPTT (s)	A PT (s)
DMSO ^1^	39.0	11.1	**24**	41.7	11.5
**21**	74.3	22.0	**25**	63.1	12.8
**22**	50.7	19.9	**26**	40.5	11.7
**23**	65.8	20.9	Heparin ^2^	34 (30 µM)	85 (30 µM)

^1^ DMSO was used as the negative control. ^2^ The molecular weight of heparin used as positive control in our experiments is 3500. aPTT: activated partial thromboplastin time, PT: prothrombin time.

**Table 6:** In vitro anticoagulant activity of amidino *N*^2^-thiophene compounds **21**–**23**.

Compound No.	Dose	aPTT (s)	PT (s)	PT (INR)
Control	saline	23.6 ± 0.6	12.4 ± 0.4	1.00
**21**	5 µM	24.1 ±0.6	12.8 ± 0.7	1.08
	10 µM	30.8 ± 0.5 *	14.5 ± 0.5 *	1.46 *
	20 µM	38.5 ± 0.4 *	16.7 ± 0.5 *	2.04 *
	30 µM	52.7 ± 0.7 *	19.3 ± 0.6 *	2.89 *
	40 µM	71.4 ± 0.6 *	22.7 ± 0.7 *	4.27 *
	50 µM	75.2 ± 0.5 *	23.5 ± 0.5 *	4.64
**22**	5 µM	22.9 ± 0.5	12.2 ± 0.4	0.96
	10 µM	23.7 ± 0.6	12.7 ± 0.4	1.06
	20 µM	30.2 ± 0.3 *	13.9 ± 0.3 *	1.32 *
	30 µM	32.8 ± 0.5 *	16.1 ± 0.7 *	1.87 *
	40 µM	48.6 ± 0.4 *	20.8 ± 0.5 *	3.46 *
	50 µM	51.4 ± 0.7 *	21.4 ± 0.6 *	3.70 *
**23**	5 µM	24.2 ± 0.2	12.1 ± 0.6	0.94
	10 µM	30.2 ± 0.5 *	12.5 ± 0.4	1.02
	20 µM	38.5 ± 0.7 *	14.2 ± 0.5 *	1.38 *
	30 µM	43.2 ± 0.4 *	17.4 ± 0.4 *	2.25 *
	40 µM	63.8 ± 0.6 *	20.1 ± 0.6 *	3.19 *
	50 µM	64.1 ± 0.4 *	20.2 ± 0.5 *	3.23 *
Heparin ^1^	20 µM	53.3 ± 0.5 *	21.6 ± 0.4 *	3.79 *
	30 µM	62.5 ± 0.8 *	27.2 ± 0.6 *	6.59 *

^1^ The molecular weight of heparin used as positive control in our experiments is 3500. Each value represents the means ± SEM (*n* = 5). * *p* < 0.05.

**Table 7:** In vivo bleeding time of amidino *N*^2^-thiophene compounds **21**–**23**.

Compound No.	Dose	Tail Bleeding Time (s)
Control	saline	30.8 ± 0.8
**21**	24.1 µg/mouse	49.2 ± 1.2 *
	32.1 µg/mouse	68.4 ± 1.0 *
	40.1 µg/mouse	70.4 ± 1.6 *
**22**	24.1 µg/mouse	33.1 ± 1.0 *
	32.1 µg/mouse	46.2 ± 0.6 *
	40.1 µg/mouse	48.3 ± 1.2 *
**23**	24.9 µg/mouse	42.3 ± 0.6 *
	33.2 µg/mouse	56.4 ± 1.6 *
	41.5 µg/mouse	58.2 ± 1.4 *
Heparin ^1^	140.0 µg/mouse	51.2 ± 0.8 *
	210.0 µg/mouse	68.4 ± 1.2 *

^1^ The molecular weight of heparin used as positive control in our experiments is 3500. Each value represents the means ± SEM (*n* = 5). * *p* < 0.05.

**Table 8:** Ex vivo clotting time of amidino *N*^2^-thiophene compounds **21**–**23**.

Compound No.	Dose	aPTT (s)	PT (s)	PT (INR)
Control	Saline	32.2 ± 0.8	12.8 ± 0.4	1.00
**21**	24.1 µg/mouse	48.8 ± 1.0 *	19.4 ± 0.8 *	2.71 *
	32.1 µg/mouse	65.8 ± 0.8 *	21.2 ± 0.6 *	3.36 *
	40.1 µg/mouse	69.4 ± 1.0 *	23.7 ± 1.2 *	4.39 *
**22**	24.1 µg/mouse	37.2 ± 1.2 *	15.7 ± 0.6 *	1.63 *
	32.1 µg/mouse	47.5 ± 0.6 *	19.8 ± 0.8 *	2.85 *
	40.1 µg/mouse	49.6 ± 0.8 *	20.1 ± 0.8 *	2.95 *
**23**	24.9 µg/mouse	39.8 ± 0.8 *	16.1 ± 0.5 *	1.73 *
	33.2 µg/mouse	54.2 ± 1.2 *	18.2 ± 0.7 *	2.33 *
	41.5 µg/mouse	56.4 ± 1.8 *	19.4 ± 0.6	2.71 *
Heparin^1)^	140.0 µg/mouse	63.4 ± 0.9 *	25.4 ± 0.4 *	5.18 *
	210.0 µg/mouse	75.4 ± 1.2 *	31.4 ± 0.6 *	8.62 *

^1^ The molecular weight of heparin used as positive control in our experiments is 3500. Each value represents the means ± SEM (*n* = 5). * *p* < 0.05.
